# 3D culturing as a promising strategy to enhance the angiogenic potential of adipose stem cell-derived secretome: insights into the role of miR-145-5p/ANGPT2 axis

**DOI:** 10.1186/s13287-025-04277-7

**Published:** 2025-03-28

**Authors:** G. Gerini, E. Mari, P. Pontecorvi, S. Camero, E. Romano, D. Ranieri, F. Megiorni, P. Fioramonti, A. Angeloni, C. Marchese, S. Ceccarelli

**Affiliations:** 1 Department of Experimental Medicine, Sapienza University of Rome, Viale Regina Elena 324, 00161 Rome, Italy; 2 Department of Life Science, Health and Health Professions, Link Campus University, Via del Casale di San Pio V 44, 00165 Rome, Italy; 3 Department of Sense Organs, Sapienza University of Rome, Viale del Policlinico 155, 00161 Rome, Italy; 4 Unit of Plastic Surgery “P. Valdoni”, Department of Surgery “P. Valdoni”, Sapienza University of Rome, Viale del Policlinico 155, 00161 Rome, Italy

**Keywords:** Adipose-derived stem cells (ASCs), Secretome, 3D spheroids, Angiogenesis, microRNAs

## Abstract

**Background:**

Adipose-derived mesenchymal stem cells (ASCs) represent a valid therapeutic option for clinical application in several diseases, mostly due to the paracrine activity of their secretome, exerting pro-angiogenic, antinflammatory and immunosuppressive effects. Recently, 3D culturing models has been shown to significantly influence the intrinsic characteristics of these cells, their gene expression and the secretome’s composition, thus affecting ASC paracrine effects and clinical potential. This study aims to investigate the feasibility of exploiting 3D culturing as a tool to improve ASC secretome therapeutic efficacy.

**Methods:**

ASCs were cultured in monolayers via conventional two-dimensional (2D) methods or induced to form 3D spheroids by seeding them on 96-well ultra-low attachment (ULA) plates. The phenotypical characterization of 3D-ASCs was performed through immunofluorescence analyses. The composition and angiogenic potential of 3D-ASC-derived secretome was assessed by means of protein array and functional tube formation assay, respectively. We analyzed the expression profile of 92 angiogenesis-related genes in 2D versus 3D cultures through a qRT-PCR array, and GO term enrichment analysis followed by network analysis was applied to identify the top hub genes. The expression of specific angiomiRs in 3D-ASCs and their secretome was assessed by qRT-PCR. The role of miR-145-5p was investigated through transfection with specific mimics/anti-miR.

**Results:**

3D-ASCs showed increased stemness, cell–cell and cell-ECM interactions with respect to 2D-cultured cells. 3D culturing increased the secretion of cytokines involved in the promotion of angiogenesis, resulting in improved angiogenic effects on HUVEC cells. Mechanistically, qRT-PCR array data indicated downregulation of angiopoietin-2 (ANGPT2) as a key factor in the 3D-ASC-secretome-induced angiogenesis. In addition, ANGPT2 was recognized as a predicted target of miR-145-5p, one of the angiomiRs found upregulated in 3D-ASCs. Depletion of miR-145-5p significantly altered ASC secretome angiogenic potential and ANGPT2 expression on HUVEC cells.

**Conclusions:**

All these findings corroborate our hypothesis that 3D culturing is able to positively modulate ASC gene expression and secretome composition in terms of pro-angiogenic potential. Indeed, our study contributes to shed light on the role of the miR-145-5p/ANGPT2 axis in this process, opening the way to innovative potentiation strategies to implement secretome-based therapies, with broad clinical applications.

**Supplementary Information:**

The online version contains supplementary material available at 10.1186/s13287-025-04277-7.

## Background

Adipose stem cells (ASC) are multipotent stem cells that can be isolated from the stromal vascular fraction of adipose tissue. ASCs possess three major characteristics that make them an important therapeutic tool in regenerative medicine and reconstructive therapy: unlimited capacity of self-renewal, differentiative potential and immunomodulatory ability. The latter mainly consists in a paracrine effect mediated by the release of multiple soluble factors, which include cytokines, growth factors and microRNAs (miRNAs), and extracellular vesicles (EVs), collectively referred to as "secretome" [1], and it is recognized as one of the principal mechanisms underlying ASC therapeutic potential [2].

Considering the challenges related to the in vitro culturing of ASCs, such as the practical steps necessary for cell isolation, expansion and characterization, as well as the limitations to their in vivo use (actual feasible administration, especially in terms of cost and safety issues, or risk of rejection, to name a few), the exploitation of a secondary cellular product (i.e. ASC secretome) represents an appealing strategy to develop cell-free treatments that would allow to overcome the aforementioned limitations [3].

The secretome composition is affected not only by ASC intrinsic characteristics, which essentially depend on the individual from which the cells originate, but also by in vitro culture conditions [4] and exposure to different agents. So, it is possible to act on environmental conditions to modulate the composition of ASC secretome prior to administration, with the aim of improving its clinical efficacy. This can be achieved by preconditioning the cells with specific molecules capable of modifying the levels and nature of secreted factors, or directly modulating genes of interest by silencing or transfection. As reported in previous in vitro studies, various priming strategies can modulate ASCs therapeutic potential [5–7], eventually increasing their beneficial paracrine effect on the surrounding microenvironment.

It is widely known that in vitro monolayer (2D) culture conditions do not accurately represent the complexity of in vivo tissues: cells are in different microenvironmental and physical conditions, and cell morphology and function of 2D cultures do not reflect their in vivo counterparts [8]. To overcome the limitations of 2D cultures, research has recently focused on developing three-dimensional (3D) cell culture systems, such as spheroids, to be employed in many research fields, ranging from oncology to drug screening and regenerative therapy [9]. Indeed, culturing ASCs in a 3D spheroid configuration has been proposed as an alternative strategy to enhance their therapeutic potential. The spheroid configuration allows to more closely recapitulate the physiological 3D microenvironment than conventional 2D cultures and determines a significant modification of the cell microenvironment. This can influence both ASC viability and survival (through the establishment of cell–cell or cell-ECM interactions) and ASC paracrine effects (through the enhanced secretion of bioactive molecules, such as anti-inflammatory mediators and pro-angiogenic factors), ultimately leading to a more powerful therapeutic effect [10].

However, 3D culture methods are able to induce profound transcriptomic changes that could determine different biological effects of ASCs and their secretome on target cells, and controversial data have been obtained applying 3D culture in different cellular contexts, indicating that a deep characterization of ASC spheroids is mandatory in order to optimize the production of enhanced cell-free products with specific characteristics for each disease model application.

ASCs have been previously shown to exert pro-angiogenic and anti-inflammatory effects also through the secretion of specific miRNAs, a class of short, endogenous, noncoding RNAs that regulate gene expression through complementary binding to 3’ UTR of target mRNAs [11]. MiRNAs post-transcriptionally regulate several cellular processes in stem cells, such as proliferation and differentiation [12, 13]. As previously mentioned, ASC secretome contains free or EVs-bound miRNAs that can be delivered and internalized by target cells, where they induce specific changes in gene expression, thus conveying ASC paracrine effects. In this context, the function of specific miRNAs—referred to as “angiomiR”—in the regulation of angiogenesis is particularly relevant. Pro-angiomiRs promote angiogenesis by targeting inhibitors of the angiogenic signaling pathways, while anti-angiomiRs impair angiogenesis by targeting its positive regulators [14]. Previous studies suggest that the enrichment of ASC secretome with pro-angiogenic miRNAs may potentiate its paracrine activity in the context of tissue repair [15, 16]. So, the full characterization of ASC secretome in terms of miRNA expression and relative biological function on target cells is recently gaining attention in the attempt to select specific miRNAs to be enriched to obtain a powerful cell-free therapeutic option.

In this study, we aimed to deeply characterize the advantages of 3D culture systems for the generation of an enhanced secretome. Moreover, we sought to investigate the molecular mechanisms underlying the secretome potentiation strategy based on 3D culture conditions, by evaluating the potential role of secreted miRNAs. The final goal will be to validate novel approaches that can efficiently translate cell-free treatment into clinics, thus contributing to improve the outcome of secretome-based therapies in humans. Indeed, investigating the angiogenic effect of ASC secretome is of particular interest in the field of regenerative medicine, since several studies are exploring the use of ASCs and their secretome in various ischemic conditions, such as ischemic heart disease, critical limb ischemia, and wound healing [17, 18]. Promising outcomes have been observed in preclinical trials using animal models of ischemic diseases. So, our strategy to improve the angiogenic effect of ASC secretome might represent a successful way to foster its clinical use for the treatment of severe ischemic diseases.

## Methods

### Adipose-derived stem cells (ASCs) isolation and culture

Liposuction aspirates of healthy donors who underwent elective plastic surgery were transferred to the laboratory and processed under sterile conditions within 24 h. Isolation of ASCs was performed as previously described [19]. Briefly, liposuction aspirates were washed with sterile phosphate-buffered saline (PBS; Aurogene, Rome, Italy) containing 2% penicillin/streptomycin and minced. The extracellular matrix was digested with 0.075% collagenase Type I (Gibco, Paisley, UK) for 30–60 min at 37 °C and 5% CO_2_. The suspension was filtered to remove debris and centrifuged for 5 min at 2000 rpm. The pellets of stromal vascular fraction (SVF) containing ASCs were washed with PBS, then resuspended in the culture medium and transferred to a T75 culture flask coated with collagen Type IV (Sigma-Aldrich, Milan, Italy). ASCs were cultured as a monolayer in DMEM-Ham’s F-12 medium (vol/vol, 1:1) (DMEM/F12; Gibco) supplemented with 10% FBS, 100 U/mL penicillin, 100 mg/mL streptomycin, and 2 mM L-glutamine, indicated as 2D-ASCs and maintained in a 5% CO_2_ incubator at 37 °C in a humidified atmosphere, with medium change twice a week. When reaching 80–90% confluence, cells were detached with 0.5 mM EDTA/0.05% trypsin (Euroclone, Milan, Italy) for 5 min at 37 °C and then replated. Cell morphology was evaluated by phase contrast microscopy. Experiments were conducted between passage numbers 3 and 6, unless otherwise specified. Absence of mycoplasma contamination was confirmed by PCR with specific primers.

### ASC spheroids formation

ASCs were expanded in DMEM-F12 medium in T75 flasks to 80–100% confluency, then collected by trypsinization, counted, and resuspended in DMEM-F12 medium to obtain a final cell density of 1 × 10^5^ cells/mL. Then, cells were induced to form spheroids by seeding them on 96-well ultra-low attachment (ULA) plates. Briefly, 100 μL of cell suspension containing 1 × 10^4^ cells were seeded in each well of ULA 96-well U-bottom plates (faCellitate BIOFLOAT™ 96-well plate). Plates were cultured for 5 days without medium changes under standard cell culture conditions. ASC spheroids (henceforth referred to as 3D-ASCs) were then collected by centrifugation, carefully resuspended and used for further experiments.

### Collection of ASC-conditioned medium (ASC-CM)

2D- and 3D-ASCs, under the different experimental conditions, were maintained in DMEM-F12 medium in T75 flasks or in ULA plates, respectively, with medium change twice a week. After 48 h from the last medium change, each supernatant was collected, centrifuged for 10 min at 1500 rpm, filtered to eliminate any debris and indicated as ASC conditioned medium (ASC-CM). ASC-CMs were stored at -20 °C for further experiments.

### Cell transfection

ASCs were plated in a 6-well plate at a density of 7 × 10^4^ cells/well. After 24 h of adherent growth, miR‐145‐5p was overexpressed or inhibited by transfection with miR‐145‐5p mimics or anti‐miR‐145‐5p, both purchased from Sigma Aldrich. Cells transfected with mimics‐NC or anti‐NC (Sigma Aldrich) were used as negative controls. All the transfections were performed in triplicate with DharmaFect Duo transfection reagent (GE Healthcare Dharmacon, Inc., Lafayette, CO, USA), according to the manufacturer’s instructions. After 72 h, cells were harvested for further experiments and the ASC-CM was obtained and stored as previously described. The transfections were repeated at least three times.

Transfection with miR‐145‐5p mimics, anti‐miR‐145‐5p and the respective NC controls were performed as described above also on human umbilical vein endothelial cells (HUVECs). HUVECs were purchased from the American Type Culture Collection (ATCC, Rockville, MD, USA), cultured in endothelial cell medium (ECM; ScienCell, USA) supplemented with 5% FBS (ScienCell, USA), 1% endothelial cell growth supplement (ECGS; ScienCell, USA), and 1% penicillin/streptomycin solution (P/S; ScienCell, USA), and plated in a 6-well plate at a density of 1 × 10^5^ cells/well prior to transfection.

### Immunofluorescence (IF) analysis

IF on 2D-ASCs was performed as previously described [20]. Briefly, cells grown on coverslips onto 24-well plates were fixed in 4% paraformaldehyde (PFA) for 30 min at room temperature (RT), followed by treatment with 0.1 M glycine (Sigma-Aldrich) in PBS for 20 min and with 0.1% Triton X-100 (Sigma-Aldrich) in PBS for additional 5 min to allow permeabilization. Cells were then assayed for the expression of specific markers by incubation with primary/secondary antibodies, and the coverslips were mounted onto a labeled slide. IF on 3D-ASCs was performed as previously described [21]. Briefly, culture medium was aspirated from the wells of ULA plates, leaving the spheroids at the bottom. Spheroids were fixed by incubating with 4% PFA at 4 °C for 15 min. Then, the plate was swirled by hand and placed back at 4 °C for another 15 min. After fixation, 3D-ASCs were transferred into a 2 mL microcentrifuge tube, using a P1000 tip with the tip cut off at the end to prevent mechanical disruption of the spheroids. Spheroids were pelleted by centrifugation at 2300 × g for 5 min, the pellet was washed twice with 0.1% TBSTX and then incubated with 0.5% TBSTX on rotator at RT for 30 min to allow permeabilization. After blocking for 1 h with 2% BSA, and incubation with primary/secondary antibodies, spheroids were resuspended in 50 μL PBS with a P200 with the end of the tip cut off, pipetted onto a labeled slide and covered with a coverslip. 2D- and 3D-ASCs were incubated with the following primary antibodies: CD29 (303001; 1:100 dilution; BioLegend, San Diego, CA, USA), CD166 (397802; 1:20 dilution; BioLegend), Vimentin (M0725; 1:100 dilution; Dako-Agilent Technologies, Santa Clara, CA, USA), cMyc (sc-40; 1:20 dilution; Santa Cruz Biotechnology, Dallas, TX, USA), Sox-2 (sc-365964; 1:20 dilution; Santa Cruz Biotechnology), E-cadherin (sc-21791; 1:20 dilution; Santa Cruz Biotechnology), Collagen 1a1 (sc-293182; 1:20 dilution; Santa Cruz Biotechnology), VEGF (sc-7269; 1:20 dilution; Santa Cruz Biotechnology). After washing, primary antibodies were visualized using FITC-conjugated goat anti-mouse IgG (Cappel Research Products, Durham, NC, United States) or Texas Red-conjugated goat anti-rabbit IgG (Jackson ImmunoResearch Laboratories, West Grove, PA, United States). Actin cytoskeleton stress fibers were visualized by incubation with TRITC-Phalloidin (P1951; 1:100 dilution; Sigma-Aldrich). Nuclei were visualized using 4’, 6-diamidino-2-phenylindole dihydrochloride (DAPI) (Sigma-Aldrich). Nonspecific fluorescence was determined by omitting the primary antibody. The single stained and merged images were acquired with a Zeiss ApoTome microscope (10 × or 40 × magnification) using the Zen software (Carl Zeiss, Jena, Germany).

### Western blot (WB) analyses

WB was performed as described in Additional file 1: Supplementary Materials and Methods.

### Cytokine array

ASC-CMs obtained as previously described from 2D/3D cultures were tested for protein secretion by Human XL Cytokine Array Kit (ARY005B; R&D Systems, Minneapolis, MN, USA). Membranes were treated and analyzed according to the manufacturer's protocol. Densitometric analyses were performed with Quantity One Program (Bio-Rad Laboratories S.r.l., Segrate, MI, Italy).

### Enzyme-linked immunosorbent assay (ELISA)

ELISA was performed as described in Additional file 1: Supplementary Materials and Methods.

### Tube formation assay

Tube formation assays were conducted as follows. Frozen Matrigel (Becton, Dickinson & Co., Franklin Lakes, NJ, USA) was thawed overnight at 4 °C, and all experimental equipment was cooled to − 20 °C before use. Matrigel (diluted 1:1 in PBS) was transferred to a 24-well plate and solidified by incubation at 37 °C and 5% CO_2_ for 30 min before seeding of the cells. HUVECs (5 × 10^5^ cells/well), transfected or not with miR‐145‐5p mimics, anti-miR-145-5p and the respective NC controls, were then seeded on Matrigel and treated or not with ASC-CMs, diluted 1:1 with basal ECM. HUVECs treated with PBS diluted 1:1 with basal ECM were used as a negative control. After 12 h, tube formation of HUVECs was observed and photographed using a Leica inverted microscope coupled with a digital camera. Tube formation assays were repeated in triplicate. The total tube length and the number of branches formed by the cells were quantified using the Angiogenesis Analyzer plug-in developed for the ImageJ software.

### Quantitative real-time PCR (qRT-PCR)

Total RNA from 2D-ASCs, 3D-ASCs and HUVEC cells was extracted using TRIzol reagent (Invitrogen, Milan, Italy). Quantity and quality of the extracted RNA were assessed by NanoDrop (Thermo Fisher Scientific, Monza, Italy). Quantitative real-time PCR assays (qRT-PCR) were conducted in triplicate on a StepOnePlusTM Real-Time PCR System (Applied Biosystems, Foster City, CA). The abundance of VEGFa and ANGPT2 was quantified using the appropriate TaqMan gene expression assay probes (Applied Biosystems). GAPDH mRNA was used as endogenous control. For miRNA detection, 40 ng of RNA were retrotranscribed with specific primers (Thermo Fisher Scientific). Expression of miR-17-5p, miR-21-5p, mir-34a-5p, miR-145-5p and miR-200c-3p was analyzed by using sequence-specific TaqMan MicroRNA Assays (Applied Biosystems). Nuclear RNA (U6) levels were used as an internal control (Applied Biosystems).

### PCR array

Gene expression quantification of genes involved in angiogenesis was performed using the TaqMan® Array Human Angiogenesis, Fast 96-well Plate (Applied Biosystems) by qRT-PCR. The panel of assays designed by the manufacturer allowed the evaluation of four reference genes (RNA, 18S ribosomal (18S), glyceraldehyde-3-phosphate dehydrogenase (GAPDH), hypoxanthine phosphoribosyltransferase 1 (HPRT1), and glucuronidase, beta (GUSB)) as endogenous controls, and 92 angiogenesis-related genes. The reactions were performed in duplicate with 25 ng of cDNA on a QuantStudio Real-Time PCR System (Applied Biosystems), according to the manufacturer's instructions. Fold changes in expression between 2D- and 3D-ASCs were determined with the 2^−ΔΔCT^ method. For normalization of gene quantification data, the geometric mean of multiple reference genes was used.

### Bioinformatic analysis of differentially expressed genes (DEGs)

To analyze the biological roles of genes, a gene ontology (GO) enrichment analysis of DEGs was performed using the Metascape database (https://metascape.org/). The protein–protein interaction (PPI) network was constructed from the Search Tool for the Retrieval of Interacting Genes/Proteins Information (STRING) database (https://string-db.org/). Briefly, DEGs were uploaded to the STRING database, and the result with an interaction score higher than 0.7 (high confidence) was visualized and edited with Cytoscape software (version 3.10.2). Furthermore, significant modules were detected through the MCODE (Molecular Complex Detection) plugin in Cytoscape based on the constructed PPI networks with the criteria of degree cut-off = 2; node score cut-off = 0.2; K-Core = 2; maximum depth = 100. Cytoscape software was then applied to analyze the hub genes, which are important nodes with many interaction partners. We utilized the CytoHubba plugin in Cytoscape to find hub genes and employed the maximal clique centrality (MCC) calculation method. The intersecting genes derived using this algorithm represent key candidate genes with important biological regulatory functions.

### Screening of potential targets for miR-145-5p

A combination of three online databases, TargetScan, miRDB, and miRTarBase, was used to predict the target genes of miR-145-5p. The 79 overlapping target genes were compared with the 27 differentially expressed genes (DEGs) obtained from the PCR array. The intersections between the predicted targets from the three databases and between predicted genes and DEGs were displayed through Venn diagrams, generated using the web-based tool Venn (http://bioinformatics.psb.ugent.be/webtools/Venn/).

### Statistical analyses

Data were analyzed on Prism 10.0 (GraphPad Software, La Jolla, USA) and are shown as mean ± SD from three independent experiments conducted in triplicate. Two-tailed unpaired Student’s t test was used for statistical analysis. *P* values < 0.05 were considered statistically significant. DEGs were identified via fold change filtering using a cut-off of absolute fold change > 2.

## Results

### Characterization of 2D versus 3D ASC culture conditions

**Fig. 1:**
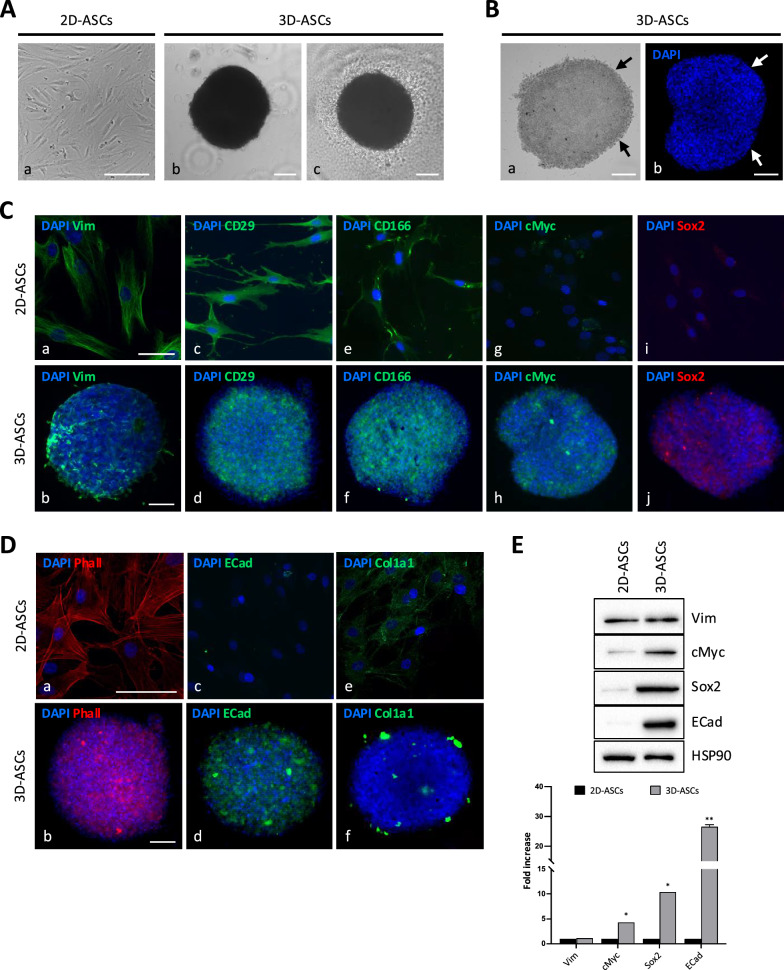
Characterization of 3D-cultured ASC spheroids. **A** Representative light microscopy images of 2D-ASCs (a) and 3D-ASCs at 72 h (b) or at 7 days (c) of sphere culturing. **B** Representative phase contrast microscopy image (a) and fluorescence image (b) showing 3D-ASC morphology. Arrows indicate elongated cells at the edge of an ASC spheroid. **C** Representative fluorescence images showing the expression of the mesenchymal marker Vimentin (green; a, b), the ASC-related markers CD29 (green; c, d) and CD166 (green; e, f), and the pluripotency genes cMyc (green; h, i) and Sox2 (red; j, l) in 2D- and 3D-ASCs. **D** Representative fluorescence images showing organization of the F-actin cytoskeleton (red; a, b), expression of E-cadherin (green; c, d) and presence of the extracellular matrix protein Col1a1 (green; e, f) in 2D- and 3D-ASCs. DAPI, 4’,6-diamidino-2- phenylindole. Scale bars, 100 μm. **E** Western blot analysis of Vimentin, cMyc, Sox2 and E-cadherin protein expression in 2D- and 3D-ASCs. HSP90 was used as internal control. The intensity of the bands was evaluated by densitometric analysis, the values were normalized and reported in graph as fold increase with respect to 2D-ASCs. *P < 0.05, **P < 0.01 *vs* 2D-ASCs

In this work, ASCs isolated from subcutaneous adipose tissue of healthy donors were used to prepare 3D spheroids (3D-ASCs). After trypsinization, a cell suspension containing varying amounts of ASCs was transferred into ultra-low attachment (ULA) plates. The sedimented cells adhered to each other, forming 3D spheroids rather than adhering to the plate surface. The morphology of ASC spheroids was compared with 2D-cultured ASCs. Light microscopy images (Fig. 1A) showed the typical spindle-shape morphology of ASCs in standard 2D culture conditions (2D-ASCs, panel a), while 3D-ASCs displayed a 3D organization of cells into a regular round shape (panel b). It is known that the spheroid sizes can be varied by modulating the cell seeding densities. So, as reported in the Additional File 1: Supplementary Results, we set the optimal conditions of cells amount per well and culture period to obtain a relatively homogeneous size distribution of spheroids (Figure S1). As shown in Fig. 1A panel c, after 7 days of 3D culturing some cells shed down from the spheroids and spread over the bottom of the wells, although not determining a significant reduction in spheroids’ size. Spheroids’ diameter showed a progressive reduction during more prolonged times of culture (data not shown), so we performed all the experiments between 3 and 7 days of culture to ensure size homogeneity. The average diameters, areas and perimeters of 3D-ASCs, prepared with a cell seeding density of 1 × 10^4^ cells per well and cultured for 5 days, were 511.9 ± 62.3 μm, 1728.7 ± 179.5 μm and 242,539.2 ± 34,532.4 μm^2^, respectively (n = 20 batches). Phase contrast and fluorescence microscopy were performed to better evaluate spatial distribution of cells in 3D-ASCs (Fig. 1B). In line with previous observations [22], it is possible to identify an outer layer of the spheroid when cells are more densely packed and elongated (Fig. 1B, arrows in panels a, b), and an inner layer formed by more dispersed and rounded cells.

The assembled ASC spheroids were then harvested and characterized by immunofluorescence analysis. We first assessed that 3D culturing would not affect ASC phenotype and stemness. We checked the expression of the mesenchymal marker Vimentin (Fig. 1C, panels a, b) and of the ASC surface markers CD29 (panels c, d) and CD166 (panels e, f), in 2D and 3D culture conditions, demonstrating that ASC within spheroids were able to maintain the same peculiar mesenchymal feature and immunophenotype characteristics expressed by 2D-cultured cells.

Some interesting studies showed the ability of 3D culture to boost stem cells pluripotency [23, 24]. So, we also evaluated the stemness markers cMyc (Fig. 1C, panels g, h) and Sox2 (panels i, j), demonstrating their increased expression in 3D-ASCs compared to adherent cells, in line with previous reports suggesting the upregulation of stemness-related transcriptional factors as a specific response to 3D culturing conditions [25].

We also confirmed previous data indicating a significant alteration of cytoskeleton organization in spheroids, as an adaptation to the 3D cellular environment. Indeed, while 2D-cultured ASCs displayed a cytoskeletal organization mainly represented by stretched stress fibers (Fig. 1D, panel a), 3D spheroids showed a loose F-actin cytoskeleton (panel b). 3D-ASCs displayed a distinct expression of the adhesion molecule E-cadherin (Fig. 1D, panel c), which is not expressed in their 2D counterparts (panel d). Such evidence is in line with the known role of E-cadherin in cell–cell interactions during MSC spheroid formation [26]. We then analyzed the expression of type I collagen, the most prevalent ECM component providing structural support to 3D tissues. The presence of aggregates of collagen within the spheroids (Fig. 1D, panel f), compared with the barely inconsistent expression of the protein in 2D culture (panel e) confirmed the ability of 3D-ASCs to establish an ECM network.

The expression of Vimentin, cMyc, Sox2 and E-cadherin in 2D- and 3D-ASCs was also assessed by WB analysis (Fig. 1E). In line with the results obtained by IF, we confirmed a similar expression of the mesenchymal marker vimentin in both 2D- and 3D-ASCs (1.2-fold, *P* = 0.51), while the stemness markers cMyc and Sox2 were more expressed in 3D-cultured spheroids (4.3-fold, *P* = 0.049 and 10.4-fold,* P* = 0.012 *vs* 2D-ASCs, respectively). E-cadherin, as previously assessed, was expressed almost exclusively in 3D-ASCs (26.6-fold, *P* = 0.007).

### Effect of 3D culturing on cytokines expression in the ASC secretome

**Fig. 2:**
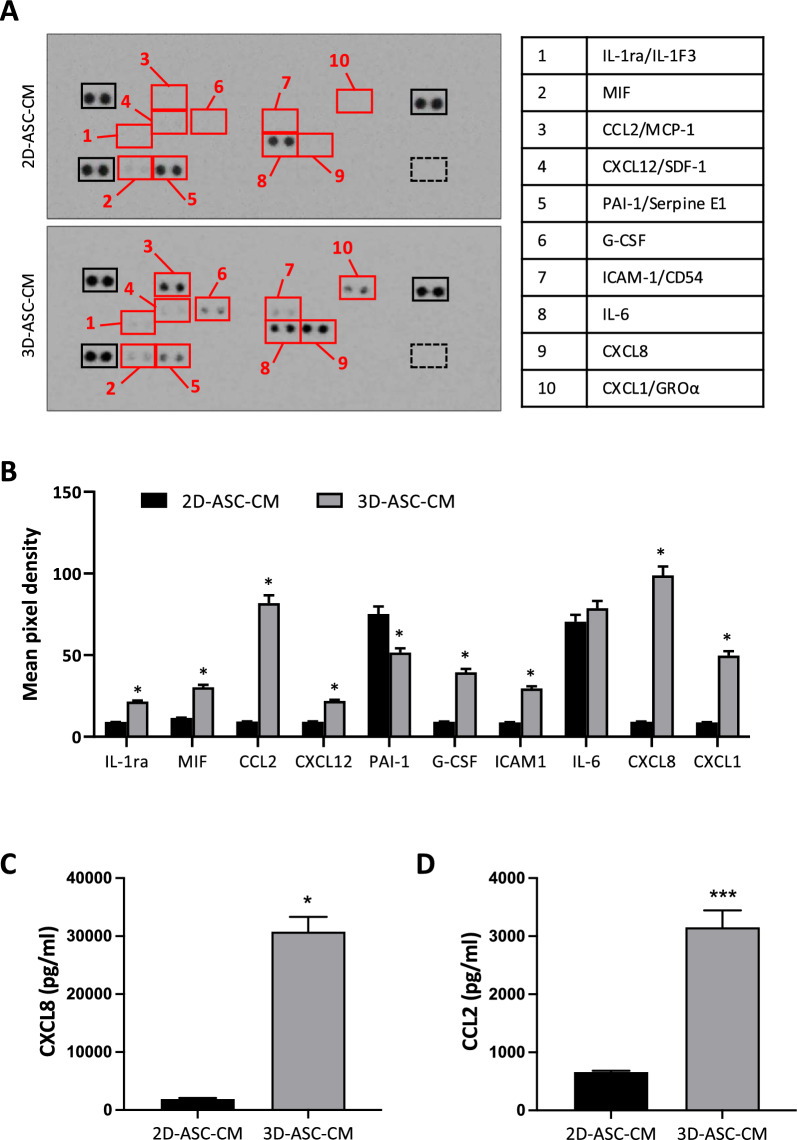
Cytokine expression profile in the secretome of 3D-cultured ASC spheroids. The expression of 36 cytokines and chemokines was assessed in the conditioned medium of 2D-cultured ASCs (2D-ASC-CM) or 3D spheroids (3D-ASC-CM) using a Protein Profiler Human Cytokine Array. ASC-CMs of three independent experiments were mixed and subjected to the assay. **A** Representative images of cytokine antibody array. The signal produced is proportional to the amount of analyte bound. Red boxes indicate the differentially expressed cytokines, reported in the table on the right. Black boxes represent positive controls; dashed black boxes represent negative controls. **B** Mean pixel density measurement obtained by image analysis software, reporting a relative quantification of the levels of differentially expressed cytokines in 2D-ASC-CM and 3D-ASC-CM. Mean ± SD of two repeats on membrane is reported in the graph. **C, D** CXCL8 and CCL2 concentrations in the conditioned medium of 2D-cultured ASCs (2D-ASC-CM) or 3D spheroids (3D-ASC-CM), measured using specific ELISA kits. *P < 0.05, ***P < 0.005 *vs* 2D-ASC-CM

In recent years, the predominant role of cytokines and their receptors in tissue repair and regeneration processes has been highlighted. Increasing evidence supports that the therapeutic effect of stem cells, especially ASCs, is mainly mediated via the secretion of cytokines, chemokines and growth factors. Some reports suggest that cell aggregation into spheroids could enhance paracrine secretion of these molecules [27, 28]. Therefore, we set out to evaluate the expression profile of human cytokines in the secretome of 3D-ASCs compared to 2D-ASCs. Conditioned medium of cells cultured either in 2D and 3D conditions was collected and analyzed using a protein array capable of detecting a panel of 36 cytokines, chemokines and soluble mediators. This analysis revealed that 3D culturing is able to induce the release of some mediators barely expressed in ASCs cultured in monolayers, namely IL-1ra, CCL2, CXCL12, G-CSF, ICAM-1, CXCL8 and CXCL1, and to modulate the secretion of other factors, such as MIF, PAI-1 and IL-6 (Fig. 2A). As reported in the graph (Fig. 2B), the most upregulated cytokines in 3D-ASC-CM were CXCL8 (10.8-fold, *P* = 0.023), CCL2 (8.8-fold, *P* = 0.025), CXCL1 (5.7-fold, *P* = 0.026) and G-CSF (4.3-fold, *P* = 0.027). To further confirm the results obtained through cytokine array, the expression of CXCL8 and CCL2 was measured by means of ELISA assay. As reported in the graphs (Fig. 2C, D), the concentrations of CXCL8 and CCL2 in 3D-ASC-CM were significantly increased with respect to those found in 2D-ASC-CM (16.3-fold, *P* = 0.038 and 4.8-fold, *P* = 0.004, respectively), thus confirming that the ability of ASCs to release these cytokines was enhanced by 3D culture conditions. Given the known role of these factors in the promotion of angiogenesis, we hypothesized that 3D culturing would increase the angiogenic potential of ASC secretome. This idea was strengthened by the observation that PAI-1, which is thought to play an important role in the inhibition of endothelial cells migration and angiogenesis, was significantly downregulated in the secretome obtained from 3D spheroids (0.7-fold).

### Effect of 3D culturing on the angiogenic potential of ASC secretome

The cytokine array highlighted specific changes in the expression and secretion of some pro-angiogenic factors elicited by 3D microenvironmental conditions. In light of these results, we hypothesized that 3D culture could significantly improve the angiogenic potential of ASC secretome with respect to 2D standard culture method.

**Fig. 3:**
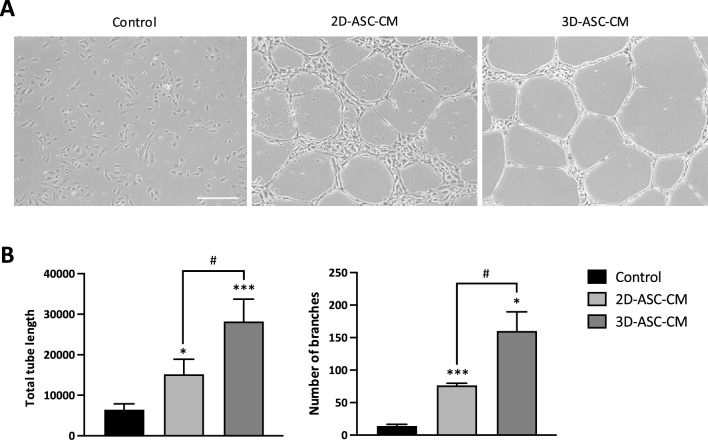
Effect of 3D culture on the ability of ASC secretome to induce angiogenesis. **A** Representative images of tube formation assay on HUVEC cells after 12 h of treatment with PBS (Control), secretome obtained from 2D cultured cells (2D-ASC-CM) or secretome obtained from 3D spheroids (3D-ASC-CM). Scale bar, 200 μm. **B** Quantification of the total tube length and number of branches. *P < 0.05, ***P < 0.005 *vs* Control; ^#^*P* < 0.05 *vs* 2D-ASC-CM

To investigate to which extent 3D-ASC-CM can promote angiogenesis, we performed a functional assay on human endothelial cells (HUVECs). The results of Matrigel-based tube formation assay (Fig. 3A) showed that, compared to PBS, the secretome obtained from 2D-cultured cells (2D-ASC-CM) significantly enhanced the angiogenic capacity of HUVECs in terms of tube length (2.4-fold, *P* = 0.043) and number of branches (5.5-fold *P* = 0.003), as previously reported in literature [29]. Interestingly, the angiogenic potential was further increased using a secretome obtained from 3D-cultured spheroids (3D-ASC-CM). Indeed, after 3D-ASC-CM treatment we observed a 1.9-fold increase in the tube length (*P* = 0.013) and a 2.1-fold increase in the number of branches (*P* = 0.038), with respect to the values obtained with 2D-ASC-CM (Fig. 3B).

### Effect of 3D culturing on the expression profile of angiogenesis-related genes

**Fig. 4:**
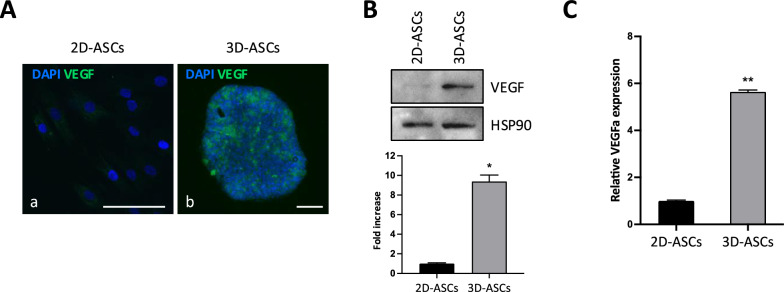
Effect of 3D culture on VEGF expression. **A** Representative immunofluorescence staining for VEGF (green) in 2D-ASCs (a) and 3D-ASCs (b). DAPI, 4’,6-diamidino-2- phenylindole. Scale bars, 100 μm. **B** Western blot analysis of VEGF protein expression in 2D- and 3D-ASCs. HSP90 was used as internal control. The intensity of the bands was evaluated by densitometric analysis, the values were normalized and reported in graph as fold increase with respect to 2D-ASCs. **C** Expression levels of VEGFa mRNA in 3D-ASCs, compared with 2D-ASCs, assessed by qRT-PCR. Each experiment was performed in triplicate and mRNA levels were normalized to GAPDH mRNA expression. Error bars represent standard deviations. *P < 0.05, **P < 0.01 *vs* 2D-ASCs

Given the information about the increased secretion of pro-angiogenic factors gathered from cytokine array and the improved angiogenic potential highlighted by tube formation assay, we decided to further analyze this aspect. To investigate the mechanisms at the basis of the increased ability of 3D-ASC-CM to promote tube formation in HUVEC cells, we analyzed the effect of 3D culturing on the expression profile of genes associated with angiogenesis. First, we assessed the expression of VEGF, a well-known potent angiogenic promoter essential for vascular neogenesis and repair. Immunofluorescence analysis revealed a significant upregulation in the expression of VEGF in 3D-cultured cells with respect to ASC monolayers (Fig. 4A, panels a, b). Such observation was confirmed through WB analysis, showing an increase in VEGF protein expression in 3D-ASCs (9.4-fold, *P* = 0.024)(Fig. 4B). Also, RT-qPCR analysis of VEGFa mRNA expression (Fig. 4C) showed a 5.6-fold increase in 3D-ASCs compared to their 2D counterparts (*P* = 0.005). Such data further endorsed the potential role of 3D culture in facilitating angiogenesis and enhancing paracrine cell communication.

**Fig. 5:**
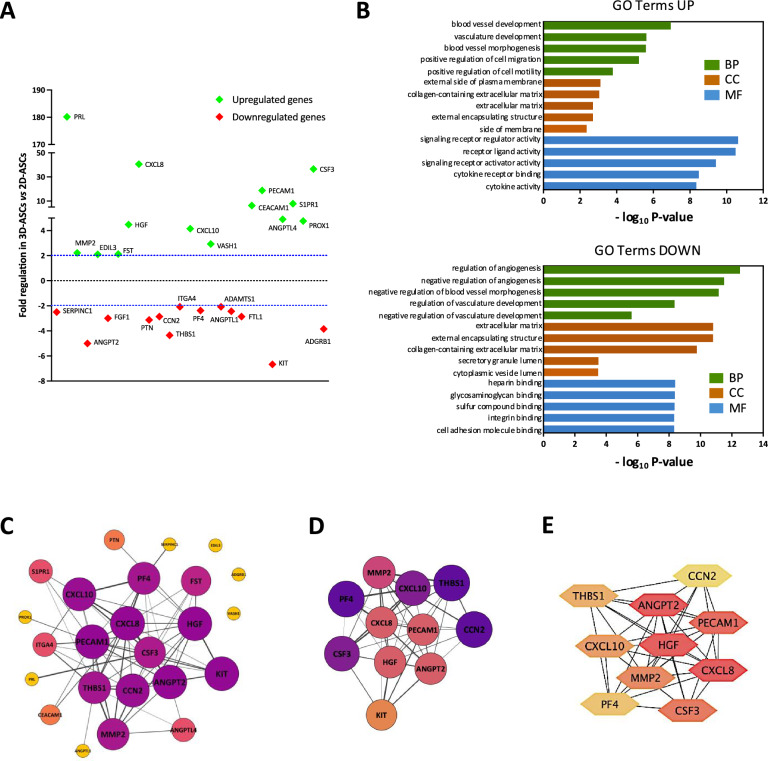
Effect of 3D culturing on the expression profile of angiogenesis-related genes. **A** Angiogenesis array gene expression data from 3D-ASCs *vs* 2D-ASCs. The upregulated and downregulated genes are shown in green and red color, respectively. Only differentially expressed genes (DEGs) with a > twofold regulation (indicated by the dotted blue lines) are depicted. **B** Gene Ontology (GO) enrichment analysis obtained by Metascape software. A false discovery rate (FDR) < 0.05 was settled as a threshold. The upregulated and downregulated genes were classified into three structural domains: biological processes (BP), cellular components (CC) and molecular functions (MF). The top 5 overrepresented GO terms in each group are displayed in the bar charts. **C** PPI network of DEGs, constructed using Cytoscape software. Nodes and font size are positively related to connectivity degree, which is further underlined by color gradient. Edge color gradient is associated with STRING combined score, computed by combining the probabilities from the different evidence channels and corrected for the probability of randomly observing an interaction. **D** The most relevant cluster of the network, visualized by MCODE in Cytoscape. Filters were based on the default parameters (degree cut-off = 2; node score cut-off = 0.2; K-Core = 2; max. depth = 100). Nodes and font size are positively related to MCODE score, which is further underlined by color gradient. **E** Top 10 hub genes screened through the maximal clique centrality (MCC) algorithm from CytoHubba. Color gradient is positively related to MCC scores

To better characterize the molecular mechanism underlying such an effect, we exploited a TaqMan Human Angiogenesis Array to profile the expression of 92 key genes involved in the biological process of angiogenesis in both 2D-cultured ASCs and 3D spheroids. The differential expression of the 81 detected genes is reported in Table S1. By applying an absolute fold regulation > 2.0 and a *P* value < 0.05 as inclusion criteria, data analysis revealed 27 differentially expressed genes (DEGs) in 3D-ASCs *vs* 2D-ASCs. As represented in the graph (Fig. 5A), 14 genes were significantly upregulated by 3D culturing, and 13 genes showed a downregulation in 3D-cultured spheroids. Interestingly, the PRL gene showed the highest increase in gene expression (180.2-fold, *P* = 0.0002) in 3D-ASCs compared to 2D-ASCs. Other significantly upregulated genes are CXCL8 (40.6-fold,* P* = 0.0001), CSF3 (36.6-fold, *P* = 0.0007), PECAM1 (18.8-fold, *P* = 0.0001), S1PR1 (7.8-fold, *P* = 0.0024), CEACAM1 (6.3-fold, *P* = 0.0005), ANGPTL4 (4.9-fold, *P* = 0.0006), PROX1 (4.8-fold,* P* = 0.0009) and HGF (4.5-fold* P* = 0.0008). All of them show a pro-angiogenic effect in different cellular contexts. On the other end, in 3D-ASCs we observed a significant decrease of several genes, such as KIT (− 6.6-fold, *P* = 0.0004), CHGA (− 5.3-fold, *P* = 0.0089), ANGPT2 (− 5.0-fold, *P* = 0.0001), THBS1 (− 4.3-fold, *P* = 0.0118) and ADGBR1 (− 3.8-fold, *P* = 0.0006), most of which have been previously identified as known inhibitors of angiogenesis. The data suggested that 3D culturing significantly alters the pattern of expression of angiogenesis-related genes.

The Gene Ontology (GO) analysis of DEGs, evaluated using the Metascape database, included three functional groups: biological processes (BP), cellular components (CC) and molecular functions (MF). Figure 5B shows the top 5 results of the GO enrichment analysis of DEGs (FDR < 0.05) in each group for upregulated and downregulated genes. In terms of biological processes (BP), the upregulated genes were mainly enriched in blood vessels development and morphogenesis, and in positive regulation of cell migration and motility. The downregulated genes were mainly enriched in negative regulation of angiogenesis and vasculature development. As for cellular components (CC), upregulated genes were mainly enriched in the external side of plasma membrane and collagen-containing extracellular matrix, while downregulated genes were mainly enriched in the extracellular matrix and vesicles lumen. In terms of molecular function (MF), upregulated genes were mainly enriched in signaling receptor activity and cytokine receptor binding and activity, while downregulated genes were enriched in heparin, integrin and cell adhesion molecules binding.

A protein–protein interaction (PPI) network including 24 nodes and 67 edges was obtained by applying STRING data to Cytoscape software (Fig. 5C). The enriched number of interactions among these DEGs is due to their biological connections as involved in angiogenesis. By using MCODE plugin, we found a cluster with 11 nodes and 44 edges (score = 8.8) (Fig. 5D). Applying the CytoHubba plugin, we detected the top 10 hub genes of the network using the maximal clique centrality (MCC) algorithm (Fig. 5E). The MCC scores of these genes are reported in Table 1. Among these hub genes, CXCL8, ANGPT2, HGF and PECAM1 can be considered as the most relevant, based on their differential modulation between 2D- and 3D-ASCs, their MCC score and their potential role in angiogenesis.

**Table 1:** Maximal clique centrality (MCC) scores of the top 10 hub genes screened from the PPI network of DEGs by using CytoHubba plug-in in Cytoscape

Gene	MCC Score
CXCL8	2456.0
ANGPT2	2454.0
HGF	2449.0
PECAM1	2419.0
CSF3	1585.0
MMP2	1446.0
CXCL10	1442.0
THBS1	859.0
PF4	841.0
CCN2	750.0

### Effect of 3D culturing on the expression profile of selected angiomiRs

**Fig. 6:**
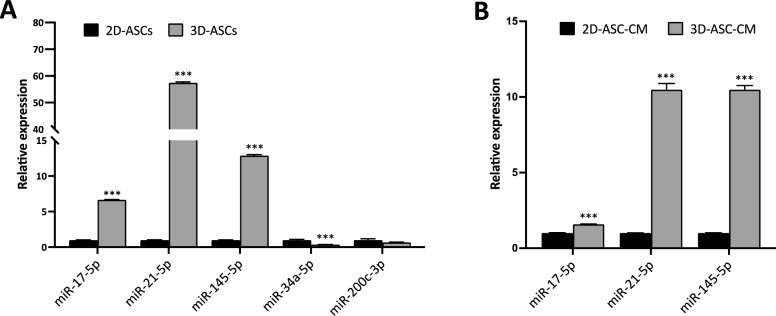
Expression of angiomiRs in ASC cells/secretome induced by 3D culture. **A** Expression levels of miR-17-5p, miR-21-5p, miR-145-5p, miR-34a-5p and miR-200c-3p in 3D-ASCs, compared with 2D-ASCs, assessed by qRT-PCR.** B** Expression levels of miR-17-5p, miR-21-5p and miR-145-5p in 3D-ASC-CM, compared with 2D-ASC-CM, assessed by qRT-PCR. Each experiment was performed in triplicate and miRNA levels were normalized to U6 expression. Error bars represent standard deviations. ***P < 0.005 *vs* 2D-ASCs or 2D-ASC-CM

It is well known the importance of miRNAs as regulators of key biological processes, including angiogenesis. Many recent studies have highlighted their key role in mediating the paracrine effect of stem cells, thanks to their active secretion both in free form and within EVs [30]. For this reason, we decided to focus on some miRNAs suggested by the literature to act as positive or negative regulators of the angiogenic process, known as “angiomiRs”. We analyzed their expression in ASC cells/secretome, specifically investigating their possible modulation induced by 3D culture. As highlighted in Fig. 6A, ASC spheroids showed a significant upregulation of miR-17-5p (6.7-fold, *P* = 0.00006), miR-21-5p (57.4-fold, *P* = 0.00009) and miR-145-5p (12.9-fold, *P* = 0.00004) with respect to 2D-cultured cells. Conversely, miR-34a-5p was significantly downmodulated in 3D-ASCs (0.4-fold, *P* = 0.0048), while miR-200c-3p did not show statistically significant variations (*P* = 0.152). Subsequently, the expression of the three upregulated angiomiRs was evaluated in the secretome of ASCs cultured in 2D and 3D, to evaluate a possible modulation in their secretion. Our results verified the presence of all these miRNAs in both 2D- and 3D-ASC-CMs. Strikingly, miR-21-5p and miR-145-5p demonstrated a consistently higher expression in 3D-ASC-CMs compared to 2D-ASC-CMs, with an approximately 10.5-fold increase (Fig. 6B, P < 0.005). Also, miR-17-5p was significantly upmodulated in 3D-ASC-CMs compared to 2D-ASC-CMs, although to a lesser extent (1.6-fold, *P* = 0.0008) (Fig. 6B). These results confirmed the modulation of various angiomiRs in 3D-ASC-CMs with respect to their 2D counterparts, highlighting a particularly significant upregulation of miR-21-5p and miR-145-5p.

### Role of miR-145-5p/ANGPT2 axis in mediating the angiogenic potential of ASC secretome

The pro-angiogenic effect of miR-21-5p has been previously demonstrated [31–33]. Conversely, miR-145-5p role in angiogenesis is still controversial, being indicated both as a potential anti-angiogenic miR [34, 35] and as a promoter of neovascularization [36, 37]. For this reason, we decided to deeply investigate the significance of miR-145-5p upregulation in 3D spheroids.

**Fig. 7:**
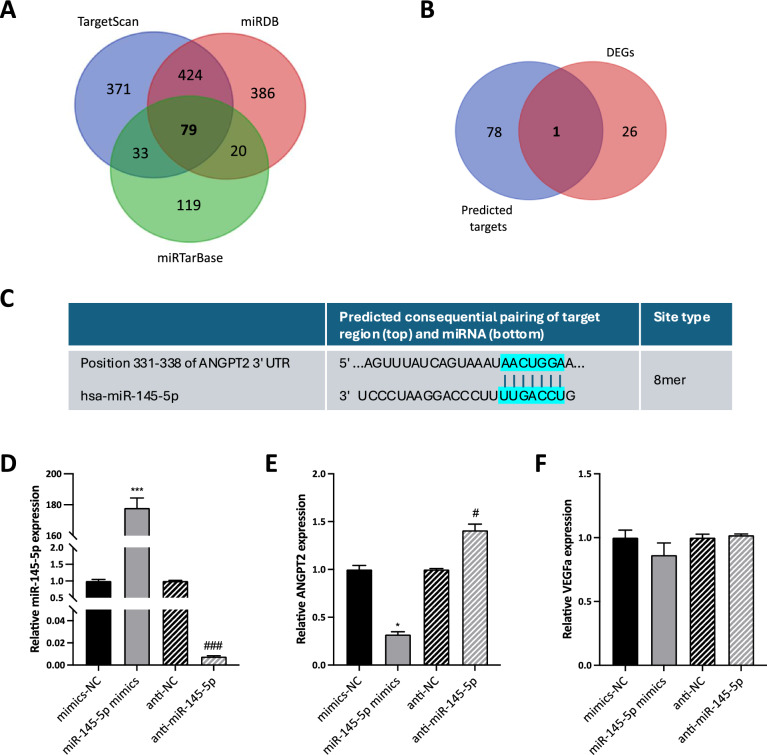
Role of miR-145-5p in the angiogenic process. **A** Venn diagram describing the potential targets of miR-145-5p based on three different databases, showing 79 overlapping genes. **B** Venn diagram describing the overlap between the screened potential targets of miR-145-5p and the DEGs obtained from the PCR array of angiogenesis-related genes. **C** Binding sites of miR-145-5p on ANGPT2 3′-UTR, predicted by a bioinformatics database. **D** Expression levels of miR-145-5p in ASCs transfected with miR-145-5p mimics, anti-miR-145-5p and the respective NC controls, assessed by qRT-PCR. **E** ANGPT2 mRNA expression in ASCs transfected with miR-145-5p mimics, anti-miR-145-5p and the respective NC controls, assessed by qRT-PCR. **F** VEGFa mRNA expression in ASCs transfected with miR-145-5p mimics, anti-miR-145-5p and the respective NC controls, assessed by qRT-PCR. Each experiment was performed in triplicate, and miRNA levels were normalized to U6 expression, while mRNA levels were normalized to GAPDH mRNA expression. Error bars represent standard deviations. *P < 0.05, ***P < 0.005 *vs* mimics-NC; ^#^*P* < 0.05, ^###^*P* < 0.005 *vs* anti-NC

First, target genes of miR-145-5p were predicted to explore the mechanisms addressed via this miRNA. Based on TargetScan, miRDB, and miRTarBase databases, 79 overlapping target genes were identified (Fig. 7A). To focus on the role of miR-145-5p in the angiogenic process, these 79 predicted targets were compared with the 27 angiogenesis-related DEGs identified through PCR array, showing only 1 overlapping gene, ANGPT2 (Fig. 7B). We visited the TargetScan website (https://www.targetscan.org/) for miRNA binding site prediction analysis, discovering that miR-145-5p binds to ANGPT2 at the 331–338 gene locus in the 3′UTR region (Fig. 7C). As shown above, this gene is significantly downmodulated in 3D-ASCs (Fig. 5A), so we could hypothesize that its inhibition is mediated by the increased levels of miR-145-5p. Furthermore, as previously demonstrated by Zhou et al., miR-145-5p directly targets ANGPT2 in gastric cancer epithelial cells [38].

So, to confirm the potential link between miR-145-5p and ANGPT2 in our cellular model, ASCs were transfected with either miR-145-5p mimics, anti-miR-145-5p or negative control miRNAs (mimics-NC and anti-NC, respectively). As shown in Fig. 7D, miR-145-5p expression was significantly upregulated in cells transfected with miR-145-5p mimics compared to those with mimics-NC (177.9-fold, *P* < 0.005), while its expression was greatly reduced in cells transfected with anti-miR-145-5p (0.01-fold, *P* < 0.005).

Besides, we confirmed that the mRNA levels of ANGPT2 were negatively regulated via miR-145-5p in ASCs. Indeed, the mRNA abundance of ANGPT2 was markedly lower in cells transfected with the miR-145-5p mimics than in those with the mimics-NC (0.3-fold, *P* = 0.042), and significantly higher in cells transfected with anti-miR-145-5p compared to those with anti-NC (1.4-fold, *P* = 0.043) (Fig. 7E). These findings indicated that miR-145-5p targets ANGPT2 in ASC cells. To exclude a concomitant anti-angiogenic effect of miR-145-5p mediated by the inhibition of VEGF expression, which has been suggested by previous reports in a different cellular model, we assessed the expression of VEGF in ASCs upon transfection with miR-145-5p mimics and anti-miR-145-5p. As reported in Fig. 7F, miR-145-5p mimics/anti-miR did not determine significant variations in VEGF expression (0.9-fold,* P* = 0.263 and 1.0-fold, *P* = 0.267, respectively).

**Fig. 8:**
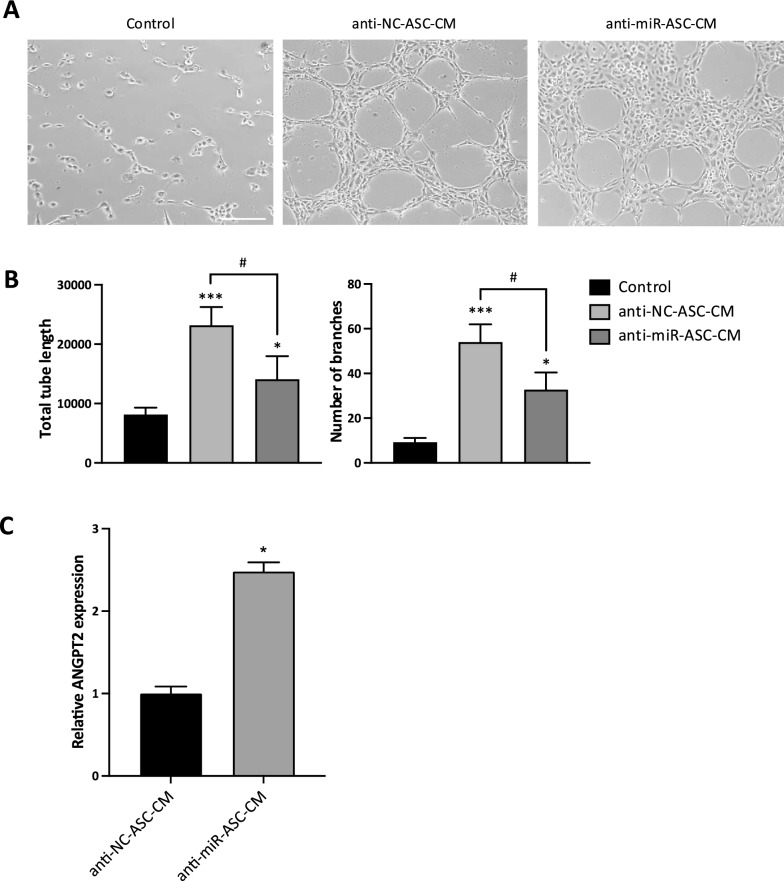
Role of miR-145-5p/ANGPT2 axis in mediating the angiogenic potential of ASC secretome. **A** Representative images of tube formation assay on HUVEC cells after 12 h of treatment with PBS (Control), secretome derived from ASCs transfected with anti-miR-145-5p (anti-miR-ASC-CM) or with anti-NC (anti-NC-ASC-CM). Scale bar, 200 μm. **B** Quantification of the total tube length and number of branches. *P < 0.05, ***P < 0.005 *vs* Control; ^#^*P* < 0.05 *vs* anti-NC-ASC-CM. **C)** ANGPT2 mRNA expression in HUVEC cells treated with secretome derived from ASCs transfected with anti-miR-145-5p (anti-miR-ASC-CM) or with anti-NC (anti-NC-ASC-CM), assessed by qRT-PCR. Each experiment was performed in triplicate, and mRNA levels were normalized to GAPDH mRNA expression. Error bars represent standard deviations. *P < 0.05 *vs* anti-NC-ASC-CM

To finally confirm the hypothesized pro-angiogenic role of miR-145-5p, we compared the angiogenic ability of the secretome generated by ASC transfected with anti-miR-145-5p (anti-miR-ASC-CM) and with the negative NC control (anti-NC-ASC-CM). By tube formation assay on HUVEC cells (Fig. 8A, B) we confirmed the angiogenic effect of ASC secretome expressing unmodified levels of miR-145-5p (anti-NC-ASC-CM), with a 2.8-fold increase in the tube length (*P* = 0.0009) and a 5.8-fold increase in the number of branches (*P* = 0.0001) with respect to Control. This effect was significantly reduced upon depletion of miR-145-5p (anti-miR-ASC-CM), with a 1.6-fold decrease in the tube length (*P* = 0.011) and a 1.6-fold decrease in the number of branches (*P* = 0.017) with respect to anti-NC-ASC-CM. The involvement of miR-145-5p/ANGPT2 axis in mediating the angiogenic potential of ASC secretome was further confirmed by the observation of a significant upregulation of ANGPT2 (2.5-fold, *P* = 0.037) in HUVEC cells upon treatment with a miR-145-5p-depleted secretome (anti-miR-ASC-CM) (Fig. 8C).

### Functional effects of miR-145-5p modulation in HUVEC cells

To validate the direct connection between miR-145-5p modulation and the angiogenesis-related functional outcomes on HUVEC cells, we compared the angiogenic ability of HUVECs transfected with miR-145-5p mimics, anti-miR-145-5p and the respective negative NC controls (mimics-NC and anti-NC), by performing tube formation assays (Figure S2A). We demonstrated a significant increase in the angiogenic potential of HUVECs expressing higher levels of miR-145-5p (miR-145-5p-mimics), with a 1.5-fold increase in the tube length (*P* = 0.003) and a 3.3-fold increase in the number of branches (*P* = 0.001) with respect to mimics-NC. Moreover, we observed a reduction in angiogenesis upon depletion of miR-145-5p (anti-miR-145-5p), with a 1.3-fold decrease in the tube length (*P* = 0.049) and a 3.0-fold decrease in the number of branches (*P* = 0.004) with respect to anti-NC (Figure S2B). Such data contribute to strengthen the hypothesis of a direct role of miR-145-5p in enhancing the angiogenic potential of HUVEC cells.

Indeed, it is known that Ang-2, the protein encoded by ANGPT2 gene, is able to compete with Ang-1 for the binding to Tie2 receptor, without causing its phosphorylation [39, 40]. So, we can hypothesize that the upregulation of Ang-2 mediated by depletion of miR-145-5p in HUVEC cells might impair angiogenesis by antagonizing Ang-1-mediated activation of Tie2. To confirm such hypothesis, we performed WB experiments on HUVEC cells transfected with anti-miR-145-5p or anti-NC control, evaluating Tie2 phosphorylation upon treatment with human recombinant Ang-1. As reported in Figure S3A, the treatment with Ang-1 was able to induce a consistent Tie2 phosphorylation, which was significantly reduced in cells transfected with anti-miR-145-5p (thereby expressing higher levels of Ang-2) (0.5-fold, *P* = 0.024) (Figure S3B). These results further strengthen the role of miR-145-5p/ANGPT2 axis in mediating the angiogenic potential of ASC secretome.

## Discussion

Over the years, ASCs have aroused growing interest in the scientific community thanks to their characteristics of unlimited capacity for self-renewal, high immunomodulatory properties, and remarkable differentiation/transdifferentiation abilities, which make them a valid therapeutic option for a variety of target diseases. At the same time, however, some limitations affect the use of ASC-based cell therapies, mainly concerning the practical steps necessary for their isolation, expansion and characterization, as well as the appropriate quality and safety controls of the final product to be injected or transplanted [3].

As it is known, most of the ASC clinical efficacy depends on the bystander effect, i.e. the modulation of the host environment through the paracrine secretion of anti-inflammatory and cytoprotective molecules. Indeed, ASCs are able to promote tissue repair/regeneration through the release of a plethora of soluble factors—including cytokines, growth factors and microRNAs—and extracellular vesicles, known collectively as "secretome". The secretome-mediated bystander effect is so robust that the use of cell-free conditioned culture medium can reproduce the same effects as ASC transplantation in some contexts [41]. Therefore, ASC secretome has emerged in recent years as a promising therapeutic resource alternative to cells, and applicable in different conditions such as wound healing and tissue repair [42, 43]. Investigating the factors that govern ASC secretome composition and its clinical efficacy is of great interest in order to develop an efficient acellular alternative. This would allow exploiting the potential of ASCs while overcoming the limitations related to the cell-based therapies.

Certainly, culture conditions have been recognized as an essential element affecting the characteristics of ASCs and their secretome. It is well known that in vitro monolayer (2D) ASC culture is not able to mimic the complexity of human adipose tissue. In recent years, three-dimensional (3D) cell culture methods that can reflect the architecture of the original tissue have been developed. Spheroids, for example, are cellular aggregates that, on a practical side, offer several advantages, including better reproducibility of in vivo conditions, lower costs, simple production processes, ease of integration into high-throughput screening and the possibility of using advanced imaging [9].

In this study, we aimed to evaluate the impact of 3D culture on ASC secretome composition and effects, comparing it with the traditional 2D culture. Firstly, we generated ASC spheroids and characterized them based on the expression of phenotypic and pluripotency markers. As expected, 3D-ASCs maintained the expression of the specific mesenchymal markers Vimentin, CD29 and CD166. In line with recent literature, we observed an increased expression of the stemness associated-proteins Sox2 and c-Myc in 3D-ASCs with respect to 2D-cultured cells, and we confirmed the ability of spheroids to drive a 3D architecture by expressing adhesion molecules (E-cadherin) and by producing their own extracellular matrix (Col1a1) [44, 45].

With respect to the secretome composition, the three-dimensional architecture stimulates ASCs to also modify their secretory profile. Indeed, we observed a significant variation in the concentration of cytokines between 2D and 3D cultures (Fig. 2). Notably, several immunosuppressive factors targeting and mobilitating the cells of the immune system, such as IL-1ra, CCL2, CXCL1, CXCL8 and G-CSF, are expressed at higher levels in the secretome obtained from ASC spheroids. Given the already high intrinsic immunomodulatory capacity of 2D ASCs, this evidence strengthens the validity of the spheroidal system as a tool to enhance this ability.

Interestingly, some of the cytokines significantly upregulated by 3D culture are reported in the literature as potentially implicated in the process of angiogenesis. Among the family of C-X-C chemokines, we observed an increase in some of those expressing pro-angiogenic properties: CXCL1, CXCL8 and CXCL12. Indeed, CXCL8 (previously called IL-8), a notoriously proinflammatory interleukin, is also able to promote angiogenesis by modulating endothelial proliferation and survival [46–48]. CXCL1, as well, has been shown to induce angiogenesis in endothelial cells, both as a paracrine and autocrine growth factor [49–51]. In line with the hypothesized enhancement of the angiogenic potential in 3D-ASC-derived secretome, we observed a significant downmodulation of the plasminogen activator inhibitor-1 (PAI-1), which has been reported to negatively influence angiogenesis in different ways: i) through downstream effects on ECM proteolysis, with subsequent inhibition of endothelial cell migration [52], ii) through direct inhibition of VEGF signaling by disruption of VEGF-VEGFR2 interaction [53]. So, the modulation of ASC secretome upon 3D culture suggests an improvement in its angiogenic potential, further supported by the evidence that the secretome obtained from 3D spheroids determined an increase in the ability of HUVEC endothelial cells to form tubular structures compared to the secretome obtained from monolayer cultures (Fig. 3).

The data obtained regarding the impact of 3D culture on angiogenesis prompted us to investigate the molecular mechanism underlying this effect, studying the differential expression of a panel of genes related to the angiogenic process in 3D- *vs* 2D-ASCs. We identified 27 differentially expressed genes (DEGs), indicated by subsequent GO enrichment analysis as involved in blood vessel development, collagen-containing extracellular matrix and regulation of angiogenesis. The PRL gene, coding for prolactin, a pituitary gland-derived hormone with roles in mammary gland development and lactation, is the most upregulated gene in 3D spheroids. Interestingly, PRL production has been also reported in mesenchymal cells, such as fibroblasts [54] and endothelial cells, which might be subject to autocrine stimulation since they also express PRL receptor [55, 56]. Some studies reported that PRL binding to PRLR might sustain angiogenic signaling through induction of STAT5 activation and VEGF secretion [57], in agreement with our observation of VEGF mRNA increase in 3D-ASCs (Fig. 4). We also observed a very consistent upregulation of CXCL8, in line with its increased secretion underlined by cytokine array on 3D-derived secretome. On the other hand, among the 13 downregulated genes we could find the ADGBR1 gene, previously known as brain-specific angiogenesis inhibitor 1 (BAI1), a member of the secretin receptor family involved in the inhibition of angiogenesis [58], and THBS1, which acts as an angiogenesis inhibitor by inducing apoptosis of endothelial cells [59]. By establishing a PPI network of DEGs and through further selection using Cytoscape plugins, we identified ANGPT2, CXCL8, HGF and PECAM1 as the most important hub genes. The role of CXCL8 in angiogenesis has been previously discussed, so its upregulation further sustains the pro-angiogenic effect of 3D culture. As for HGF and PECAM1, the first is one of the most potent mitogens for endothelial cells and is involved in vascular repair [60, 61], while the second (also known as CD31) is considered as an important marker of angiogenesis, since it is highly expressed by proliferating endothelial cells [62]. Interestingly, ANGPT2 gene encodes for angiopoietin 2 (Ang-2), an antagonist of Ang-1 that prevents the activation of its receptor Tie2 by competitive binding. Since Ang-2 is expressed at the site of vascular remodeling, its antagonism of Ang-1 action promotes vessel destabilization and potential regression, effectively determining an anti-angiogenic effect [63]. Therefore, inhibition of ANGPT2 expression could contribute to enhance the angiogenic effect of the 3D secretome.

Several studies have shown that miRNAs can be secreted, both associated with extracellular vesicles (EVs) and in a vesicle-free state, associated with proteins, such as Argonaute2 (AGO2) or high-density lipoprotein (HDL) [64]. Both vesicle-associated miRNAs and vesicle-free secreted miRNAs can be delivered to target cells, where they can regulate biological processes and eventually induce changes in gene expression [30. Therefore, secreted microRNAs can be considered as mediators of ASC paracrine effects. Previous studies demonstrated that vesicle-encapsulated miRNAs represent only a minor portion of circulating miRNAs [65], and the complete removal of proteins and vesicle-free miRNAs still represent a challenging step in EV isolation protocols [66, 67]. So, we decided to focus our analysis on the complex of secreted miRNAs.

The comprehensive characterization of ASC secretome in terms of angiomiRs and related biological function on target cells is recently gaining attention, in an attempt to select specific miRNAs to be enriched to obtain a potent cell-free therapeutic option. MiR-17-5p, part of the miR-17 ~ 92 cluster, has been shown to stimulate tumor angiogenesis by targeting anti-angiogenic proteins such as CCN2 [68]. In agreement with this study, we observed both an upregulation of miR-17-5p and a downmodulation of CCN2 in 3D-ASCs. The massive upregulation of miR-21-5p observed in 3D-ASCs and their secretome in our study is also in line with its recently demonstrated upregulation in the conditioned media of 3D-cultured umbilical cord mesenchymal stem cells, potentially accounting, together with miR-126-5p and miR-130a-3p, for their improved angiogenic potential [69]. Indeed, miR-34a has been shown to suppress angiogenesis by inducing endothelial progenitor cell senescence [70], while miR-200 family is known to inhibit angiogenesis by targeting IL-8 and CXCL1 [71]. So, our evidence of a downmodulation of miR-34a-5p and miR-200c-3p further strengthened the pro-angiogenic potential of 3D-ASC-derived secretome.

Interestingly, miR-145-5p has been shown to regulate angiogenesis in both directions, depending on the context: in normal endothelial cells or adipose stem cells, it exerts a pro-angiogenic action [36, 37], while in the context of cancer it generally plays an inhibitory role in tumor angiogenesis [34, 72]. Our results, reporting a consistent upregulation of miR-145-5p in both 3D-ASCs and 3D-ASC-derived secretome, suggest a pro-angiogenic role of this miRNA in our context, also confirmed by the reduced in vitro tube formation by HUVEC cells treated with a miR-145-5p-depleted ASC-CM. Among the potential targets of miR-145-5p, we focused on ANGPT2, since this miRNA has been previously shown to directly bind the 3′-UTR of ANGPT2 mRNA [38]. Indeed, we confirmed that ANGPT2 expression was inversely related to miR-145-5p levels in ASCs. Furthermore, our data indicated that miR-145-5p released in ASC secretome could also induce an inverse modulation of ANGPT2 expression in endothelial cells, which could partially account for its angiogenic effect. Indeed, we confirmed that overexpressing ANGPT2 in HUVEC cells by transfection with anti-miR-145-5p can counteract the activation of Tie2 signaling by Ang-1, thus strengthening the hypothesis of a competition between Ang-1 and Ang-2 [63].

This study provides a demonstration of ASC angiogenic potential and its underlying mechanisms in vitro and contribute to clarify the role of 3D culture as a key modulator of ASC secretome profile. However, our study presents some limitations. First, more detailed studies on preclinical models are needed to definitely demonstrate that ASC secretome can be considered as a therapeutic option for diseases characterized by impaired angiogenesis. Second, although many laboratory and animal studies have been performed using stem cell spheroids and their secretome, there are still very limited human clinical trials, with subsequent few information on the best administration route, dosage and timing of treatment. So, further studies are needed to prove ASC secretome therapeutic effect in the clinic.

## Conclusions

ASCs are widely exploited in the field of regenerative medicine for their differentiation capabilities and paracrine properties conveyed by their secretome. The direct influence of culture systems on the secretome composition and efficacy, combined with the ever-increasing need to bring study models closer to in vivo conditions, has led to the development of 3D models, including cellular spheroids, that can combine a more physiologically relevant microenvironment with an improved therapeutic action. This study, which thoroughly characterizes ASC spheroids and investigates the composition and angiogenic potential of their secretome, contributes to shed light on its possible use as an alternative tool to be employed in tissue repair/regeneration. Based on our results, we suggest that 3D culture is able to potentiate the pro-angiogenic effect of ASC secretome, and we can hypothesize a molecular mechanism underlying this effect that involves the miR-145-5p/ANGPT2 axis. Our study could open the way to innovative potentiation strategies to implement secretome-based therapies, with broad applications in various clinical fields, and in particular in those diseases characterized by severe ischemic conditions, such as ischemic heart disease. The future of ASC-derived secretome therapy in ischemic diseases is promising, yet it necessitates further research to bridge the gap between laboratory research and clinical practice.

## Supplementary Information

Additional file1 (DOCX 2938 KB)

Additional file2 (DOCX 687 KB)

Additional file3 (XLS 1075 KB)

Additional file4 (PDF 4430 KB)

## Data Availability

The data that support the findings of this study are available from the corresponding author upon reasonable request. Raw data from qRT-PCR array can be found as supplementary material (Additional file 3).
