# Supplementary material for: 3D culturing as a promising strategy to enhance the angiogenic potential of adipose stem cell-derived secretome: insights into the role of miR-145-5p/ANGPT2 axis
Source: Stem Cell Res Ther. 2025 Mar 28;16:153. doi: 10.1186/s13287-025-04277-7 (PMC11951674; doi:10.1186/s13287-025-04277-7)
Supplement: Supplementary file 1 — Additional file1 (DOCX 2938 KB) [file 13287_2025_4277_MOESM1_ESM.docx]

**3D culturing as a promising strategy to enhance the angiogenic potential of adipose stem cell-derived secretome: insights into the role of miR-145-5p/ANGPT2 axis**

**Gerini G.^a^, Mari E.^b^, Pontecorvi P.^a^, Camero S.^b^, Romano E.^c^, Ranieri D.^b^, Megiorni F.^a^, Fioramonti P.^d^, Angeloni A.^a^, Marchese C.^a^, and Ceccarelli S.^a*^**

^a^Department of Experimental Medicine, Sapienza University of Rome, Viale Regina Elena 324, 00161 Rome, Italy

^b^Department of Life Science, Health and Health Professions, Link Campus University, Via del Casale di San Pio V 44, 00165, Rome, Italy

^c^Department of Sense Organs, Sapienza University of Rome, Viale del Policlinico 155, 00161 Rome, Italy

^d^Department of Surgery "P. Valdoni", Unit of Plastic Surgery "P. Valdoni", Sapienza University of Rome, Viale del Policlinico 155, 00161 Rome, Italy

^*^Corresponding author: [simona.ceccarelli@uniroma1.it](mailto:simona.ceccarelli@uniroma1.it)

**SUPPLEMENTARY MATERIALS AND METHODS**

**Enzyme-linked immunosorbent assay (ELISA)**

The concentrations of CCL2 and CXCL8 in the ASC-CMs were determined by ELISA assays, which were performed according to the protocols described in the Human CCL2/MCP-1 (DP00) and Human IL-8/CXCL8 (D8000C) Quantikine ELISA Kits (R&D Systems, Minneapolis, MN, USA). The absorbance (450 nm) for each supernatant was analyzed by a microplate reader (Bio-Rad Laboratories S.r.l., Segrate, MI, Italy) and was interpolated with a standard curve.

**Western blot (WB) analysis**

For the characterization of 2D- and 3D-ASCs, cells were lysed in RIPA buffer and processed for WB analysis as previously described (Ceccarelli et al., 2020). Briefly, total proteins (30–100μg) were resolved under reducing conditions by 7–15% SDS-PAGE and transferred to Immobilon-FL membranes (Millipore, Billerica, MA, United States). The membranes were incubated overnight at 4 °C with primary antibodies to Vimentin (M0725; 1:1000 dilution; Dako-Agilent Technologies, Santa Clara, CA, USA), cMyc (sc-40; 1:200 dilution; Santa Cruz Biotechnology, Dallas, TX, USA), Sox2 (sc-365964; 1:200 dilution; Santa Cruz), E-cadherin (sc-21791; 1:200 dilution; Santa Cruz), VEGF (sc-7269; 1:200 dilution; Santa Cruz). HSP90 (13171-1-AP; 1:3000 dilution; Proteintech, Rosemont, IL, USA) was used as internal control.

For the evaluation of Tie2 phosphorylation, HUVEC cells, transfected with anti-miR-145-5p or with the anti-NC control as previously described, were incubated with 200 ng/ml of Recombinant Human Angiopoietin-1 Protein (923-AN-025; R&D Systems) for 15 min. Then, cells were lysed and subjected to WB as described above. The membranes were incubated overnight at 4 °C with primary antibodies to phospho-Tie2 (Y992) (AF2720, 1:1000 dilution) and Tie2 (AF313; 1:500 dilution), both from R&D Systems.

Primary antibodies were followed by the appropriate horseradish peroxidase (HRP)-conjugated anti-rabbit (1:10000 dilution; Advansta, San Jose, CA, USA), anti-mouse (1:10000 dilution; Jackson ImmunoResearch, West Grove, PA, USA) and anti-goat (1:5000 dilution; Santa Cruz) secondary antibody. Bound antibody was detected using the WesternBright ECL HRP substrate kit (Advansta) according to the manufacturer’s instructions. Densitometric analysis was performed with Quantity One Program (Bio-Rad).

**SUPPLEMENTARY RESULTS**

**Characterization of 3D ASC spheroids obtained from different cell seeding conditions**

Previous reports demonstrated that the properties of mesenchymal stem cells cultured as spheroids are influenced by the experimental conditions (1,2). So, we performed some experiments to optimize the conditions of spheroids formation and culture, in order to obtain good homogeneity and to avoid the introduction of variables related to the spheroid dimensions. To this aim, we formed ASC spheroids by the hanging drop technique using 4 different cell seeding densities: 1 x 10^4^, 2.5 x 10^4^, 1 x 10^5^ and 2.5 x 10^5^ cells for each well. The highest seeding densities (2.5 x 10^5^ and 1 x 10^5^ cells/well) has resulted in the formation of aggregates composed by spheroids of variable sizes (Figure S1, panels a and b), probably due to the excess of cells inside the drops. The intermediate cell density (2.5 x 10^4^ cells/well) still determined the formation of large spheroids, with a diameter of approximately 700 μm and irregular shapes (Figure S1, panel c). By using the lowest cell density (1 x 10^4^ cells/well) we could obtain single spheroids with regular shape and a diameter of approximately 500 μm (Figure S1, panel d). As reported in literature, spheroids larger than 500 μm in diameter are characterized by progressively limitation of oxygen perfusion, thus inducing the development of a hypoxic core that can alter cell behavior and generate a higher number of apoptotic or necrotic cells in the core, with a potential reduction of cell viability (3,4). Moreover, spheroids with a diameter of approximately 700 μm have been shown to display decreased mRNA expression of therapeutic proteins (2). For these reasons, we adopted the same cell density (1 x 10^4^ cells/well) for all the subsequent experiments involving spheroids.

**SUPPLEMENTARY REFERENCES**

1. Bartosh TJ, Ylöstalo JH, Mohammadipoor A, Bazhanov N, Coble K, Claypool K, et al. Aggregation of human mesenchymal stromal cells (MSCs) into 3D spheroids enhances their antiinflammatory properties. Proc Natl Acad Sci U S A. 2010;107(31):13724–9.

2. Chen LC, Wang HW, Huang CC. Modulation of inherent niches in 3d multicellular msc spheroids reconfigures metabolism and enhances therapeutic potential. Cells. 2021 Oct 1;10(10).

3. Ryu NE, Lee SH, Park H. Spheroid culture system methods and applications for mesenchymal stem cells. Cells. 2019;8(12):1–13.

4. Vinci M, Gowan S, Boxall F, Patterson L, Zimmermann M, Court W, et al. Advances in establishment and analysis of three-dimensional tumor spheroid-based functional assays for target validation and drug evaluation. BMC Biol [Internet]. 2012;10(1):29. Available from: http://www.biomedcentral.com/1741-7007/10/29

**Table S1. Differential expression of angiogenesis-related genes in 3D-ASCs versus 2D-ASCs**

| Gene Symbol | Gene Name | Regulation | Fold change | *P* value |
| --- | --- | --- | --- | --- |
| PRL | Prolactin | UP | 180.2 | 0.0002 |
| CXCL8 | C-X-C Motif Chemokine Ligand 8 | UP | 40.6 | 0.0001 |
| CSF3 | Colony Stimulating Factor 3 | UP | 36.6 | 0.0007 |
| PECAM1 | Platelet And Endothelial Cell Adhesion Molecule 1 | UP | 18.8 | 0.0001 |
| S1PR1 | Sphingosine-1-Phosphate Receptor 1 | UP | 7.8 | 0.0024 |
| CEACAM1 | CEA Cell Adhesion Molecule 1 | UP | 6.3 | 0.0005 |
| ANGPTL4 | Angiopoietin Like 4 | UP | 4.9 | 0.0006 |
| PROX1 | Prospero Homeobox 1 | UP | 4.8 | 0.0009 |
| HGF | Hepatocyte Growth Factor | UP | 4.5 | 0.0008 |
| CXCL10 | C-X-C Motif Chemokine Ligand 10 | UP | 4.1 | 0.0011 |
| VASH1 | Vasohibin 1 | UP | 2.9 | 0.0010 |
| MMP2 | Matrix Metallopeptidase 2 | UP | 2.2 | 0.0382 |
| EDIL3 | EGF Like Repeats And Discoidin Domains 3 | UP | 2.1 | 0.0005 |
| FST | Follistatin | UP | 2.1 | 0.0003 |
| FLT4 | Fms Related Receptor Tyrosine Kinase 4 | UP | 2.0 | 0.0003 |
| LYVE1 | Lymphatic Vessel Endothelial Hyaluronan Receptor 1 | UP | 2.0 | 0.0026 |
| PDGFB | Platelet Derived Growth Factor Subunit B | UP | 2.0 | 0.0021 |
| TGFB1 | Transforming Growth Factor Beta 1 | UP | 2.0 | 0.0001 |
| ANGPTL2 | Angiopoietin Like 2 | UP | 1.9 | 0.0003 |
| TIE1 | Tyrosine Kinase With Immunoglobulin Like And EGF Like Domains 1 | UP | 1.8 | 0.0054 |
| TYMP | Thymidine Phosphorylase | UP | 1.7 | 0.0013 |
| ITGB3 | Integrin Subunit Beta 3 | UP | 1.6 | 0.0020 |
| MDK | Midkine | UP | 1.6 | 0.0017 |
| NRP2 | Neuropilin 2 | UP | 1.6 | 0.0001 |
| TIMP3 | TIMP Metallopeptidase Inhibitor 3 | UP | 1.6 | 0.0024 |
| CD44 | CD44 Molecule (IN Blood Group) | UP | 1.5 | 0.0028 |
| CXCL12 | C-X-C Motif Chemokine Ligand 12 | UP | 1.5 | 0.0004 |
| ANG,RNASE4 | Novel Protein, ANG-RNASE4 Readthrough | UP | 1.4 | 0.0050 |
| PDGFRA | Platelet Derived Growth Factor Receptor Alpha | UP | 1.4 | 0.0073 |
| PDGFRB | Platelet Derived Growth Factor Receptor Beta | UP | 1.4 | 0.0099 |
| SERPINB5 | Serpin Family B Member 5 | UP | 1.4 | 0.0316 |
| TNF | Tumor Necrosis Factor | UP | 1.4 | 0.0048 |
| COL15A1 | Collagen Type XV Alpha 1 Chain | UP | 1.3 | 0.0121 |
| COL18A1 | Collagen Type XVIII Alpha 1 Chain | UP | 1.2 | 0.2482 |
| TGFA | Transforming Growth Factor Alpha | UP | 1.2 | 0.4883 |
| AMOT | Angiomotin | UP | 1.1 | 0.5244 |
| CXCL2 | C-X-C Motif Chemokine Ligand 2 | UP | 1.1 | 0.0644 |
| FOXC2 | Forkhead Box C2 | UP | 1.1 | 0.4883 |
| SEMA3F | Semaphorin 3F | UP | 1.1 | 0.1496 |
| KIT | KIT Proto-Oncogene, Receptor Tyrosine Kinase | DOWN | -6.6 | 0.0004 |
| CHGA | Chromogranin A | DOWN | -5.3 | 0.0089 |
| ANGPT2 | Angiopoietin 2 | DOWN | -5.0 | 0.0001 |
| THBS1 | Thrombospondin 1 | DOWN | -4.3 | 0.0118 |
| ADGRB1 | Adhesion G Protein-Coupled Receptor B1 | DOWN | -3.8 | 0.0006 |
| PTN | Pleiotrophin | DOWN | -3.1 | 0.0013 |
| FGF1 | Fibroblast Growth Factor 1 | DOWN | -3.0 | 0.0015 |
| CCN2 | Cellular Communication Network Factor 2 | DOWN | -2.9 | 0.0003 |
| FLT1 | Fms Related Receptor Tyrosine Kinase 1 | DOWN | -2.9 | 0.0002 |
| SERPINC1 | Serpin Family C Member 1 | DOWN | -2.5 | 0.0943 |
| ANGPTL1 | Angiopoietin Like 1 | DOWN | -2.4 | 0.0003 |
| PF4 | Platelet Factor 4 | DOWN | -2.4 | 0.0017 |
| ADAMTS1 | ADAM Metallopeptidase With Thrombospondin Type 1 Motif 1 | DOWN | -2.1 | 0.0006 |
| ITGA4 | Integrin Subunit Alpha 4 | DOWN | -2.1 | 0.0002 |
| HSPG2 | Heparan Sulfate Proteoglycan 2 | DOWN | -2.0 | 0.0026 |
| IL12A | Interleukin 12A | DOWN | -2.0 | 0.0029 |
| LEP | Leptin | DOWN | -2.0 | 0.0002 |
| FBLN5 | Fibulin 5 | DOWN | -1.9 | 0.0015 |
| CDH5 | Cadherin 5 | DOWN | -1.8 | 0.0022 |
| FN1 | Fibronectin 1 | DOWN | -1.8 | 0.0034 |
| TNFSF15 | TNF Superfamily Member 15 | DOWN | -1.8 | 0.0003 |
| ENPP2 | Ectonucleotide Pyrophosphatase/Phosphodiesterase 2 | DOWN | -1.7 | 0.0008 |
| KDR | Kinase Insert Domain Receptor | DOWN | -1.7 | 0.0044 |
| ANGPT1 | Angiopoietin 1 | DOWN | -1.6 | 0.0050 |
| THBS2 | Thrombospondin 2 | DOWN | -1.6 | 0.0079 |
| COL4A2 | Collagen Type IV Alpha 2 Chain | DOWN | -1.5 | 0.0003 |
| NRP1 | Neuropilin 1 | DOWN | -1.5 | 0.0021 |
| TNMD | Tenomodulin | DOWN | -1.5 | 0.0008 |
| COL4A1 | Collagen Type IV Alpha 1 Chain | DOWN | -1.4 | 0.0016 |
| IFNB1 | Interferon Beta 1 | DOWN | -1.4 | 0.0021 |
| F2 | Coagulation Factor II, Thrombin | DOWN | -1.3 | 0.0026 |
| ITGAV | Integrin Subunit Alpha V | DOWN | -1.3 | 0.0361 |
| TIMP2 | TIMP Metallopeptidase Inhibitor 2 | DOWN | -1.3 | 0.0205 |
| VEGFB | Vascular Endothelial Growth Factor B | DOWN | -1.3 | 0.0108 |
| COL4A3 | Collagen Type IV Alpha 3 Chain | DOWN | -1.2 | 0.0057 |
| GRN | Granulin Precursor | DOWN | -1.2 | 0.0132 |
| PROK1 | Prokineticin 1 | DOWN | -1.2 | 0.0050 |
| VEGFA | Vascular Endothelial Growth Factor A | DOWN | -1.2 | 0.0026 |
| VEGFC | Vascular Endothelial Growth Factor C | DOWN | -1.2 | 0.0034 |
| EPHB2 | EPH Receptor B2 | DOWN | -1.1 | 0.1278 |
| FGF2 | Fibroblast Growth Factor 2 | DOWN | -1.1 | 0.2129 |
| VEGFD | Vascular Endothelial Growth Factor D | DOWN | -1.1 | 0.0463 |

**Figure S1. Morphological characterization of ASC spheroids obtained with the hanging drop method using different cell seeding densities.** a, b) Phase contrast micrographs showing the morphology of spheroids obtained from a suspension of 2.5 x 10^5^ cells/well (a) or 1 x 10^5^ cells/well (b), showing variable diameters, irregular shape and spheroid aggregation. c) Phase contrast micrographs showing the morphology of spheroids obtained from a suspension of 2.5 x 10^4^ cells/well, showing an irregular shape and a size of approximately 700 μm. d) Phase contrast micrographs showing the morphology of spheroid obtained from a suspension of 1 x 10^4^ cells/well, with a regular shape and a diameter of approximately 450 μm. Scale bar 400 μm.

**Figure S2. Direct role of miR-145-5p in mediating the angiogenic potential of HUVEC cells. A)** Representative images of tube formation assay on HUVEC cells transfected with miR-145-5p mimics, mimics-NC control, anti-miR-145-5p or anti-NC control. Scale bar, 200 μm. **B)** Quantification of the total tube length and number of branches. **P* < 0.05, ****P* < 0.005 *vs* mimics-NC; ^#^*P* < 0.05, ^###^*P* < 0.005 *vs* anti-NC.

**Figure S3. Effect of miR-145-5p downmodulation on Ang-1-dependent Tie2 activation in HUVEC cells. A)** Western blot analysis of phospho-Tie2 and Tie2 protein expression in HUVEC cells transfected with anti-NC or anti-miR-145-5p and treated with 200 ng/ml of recombinant Ang-1 for 15 min. **B)** The intensity of the bands was evaluated by densitometric analysis, the values were normalized and reported as fold increase with respect to anti-NC. *P < 0.05 vs anti-NC.
