# Supplementary material for: 3D culturing as a promising strategy to enhance the angiogenic potential of adipose stem cell-derived secretome: insights into the role of miR-145-5p/ANGPT2 axis
Source: Stem Cell Res Ther. 2025 Mar 28;16:153. doi: 10.1186/s13287-025-04277-7 (PMC11951674; doi:10.1186/s13287-025-04277-7)
Supplement: Supplementary file 2 — Additional file2 (DOCX 687 KB) [file 13287_2025_4277_MOESM2_ESM.docx]

WB Fig. 1 UNCROPPED

WB Fig. 4 UNCROPPED

WB Fig. S3 UNCROPPED
