# Supplementary material for: 3D culturing as a promising strategy to enhance the angiogenic potential of adipose stem cell-derived secretome: insights into the role of miR-145-5p/ANGPT2 axis
Source: Stem Cell Res Ther. 2025 Mar 28;16:153. doi: 10.1186/s13287-025-04277-7 (PMC11951674; doi:10.1186/s13287-025-04277-7)
Supplement: Supplementary file 4 — Additional file4 (PDF 4430 KB) [file 13287_2025_4277_MOESM4_ESM.pdf]

MATERIAL TRANSFER AGREEMENT ("MTA")

Last updated: November 15, 2011

ATCC®

IMPORTANT! PLEASE READ CAREFULLY BEFORE SUBMITTING AN ORDER. THIS IS A CONTRACT.

This Material Transfer Agreement ("MTA") is between the DEPT. EXPERIMENTAL MEDICINE SAPIENZA UNIVERSITY of ROME, a University type organization, having its principal place of business at Viale Regina Elena, 324 Roma 00161 ("Purchaser") and the American Type Culture Collection, a District of Columbia not-for-profit corporation, having its principal place of business at 10801 University Boulevard, Manassas, VA 20110-2209 ("ATCC"). Purchaser must have an approved, current ATCC account to place an order. This MTA is effective as of the last date of execution by the parties and governs the purchase and use of all Biological Materials under the terms and conditions set forth below.

TERMS AND CONDITIONS

Definitions

"**ATCC Material**" means any materials acquired from ATCC by Purchaser as described on an ATCC Sales Order.

"**ATCC Sales Order**" means an order submitted for ATCC Material in a form and format as determined by ATCC from time to time.

"**Biological Material(s)**" means any ATCC Material, any Progeny, any Unmodified Derivatives and any of the foregoing materials contained or incorporated in Modifications.

"**CAR**" means ATCC's Customer Acceptance of Responsibility for Potentially Highly Pathogenic Biological Materials Form.

"**Commercial Use**" means the sale, license, lease, export, transfer or other distribution of the Biological Materials to a person or entity not party to this MTA for financial gain or other commercial purposes and/or the use of the Biological Material: (a) to provide a service to a person or entity not party to this MTA for financial gain; (b) to produce or manufacture products for general sale or products for use in the manufacture of products ultimately intended for general sale; or (c) in connection with proficiency testing service(s), including but not limited to, providing the service of determining laboratory performance by means of comparing and evaluating calibrations or tests on the same or similar items or materials in accordance with predetermined conditions.

"**Contributor(s)**" means any organization(s), entity(ies) and/or individual(s) providing technology and/or patent rights related to the ATCC Material, or original material to ATCC for deposit.

"**CRO**" means a third-party entity performing research under contract for Purchaser.

"**Industry Sponsored Academic Research**" means research sponsored by a for-profit organization carried out at a non-profit organization and by the non-profit organization's employees.

"**Investigator**" means the individual principal scientist or researcher, who is referenced on the applicable ATCC Sales Order, employed or retained by Purchaser and using the Biological Material(s) and for whom the related ATCC Material was obtained by Purchaser, as well as his or her individual assistants for receipt of ATCC Material as employed or retained by Purchaser and referenced on the applicable ATCC Sales Order. For the avoidance of doubt, Investigator specifically does NOT include any multi-user biological material core facility or repository or any individual employed or retained thereby (in a capacity including the use of the Biological Material). ATCC Material may not be transferred to any multi-user biological material core facility or repository or any such individual.

"**Modification(s)**" means any substance that contains and/or incorporates a significant or substantial portion of ATCC Material, its Progeny or Unmodified Derivatives. By way of nonlimiting example, Modifications include ATCC Material that has been transfected with a reporter gene, induced pluripotent stem cells and mesenchymal stem cells that were derived from ATCC Material.

"**Progeny**" means an unmodified descendant from ATCC Material, such as virus from virus, cell from cell, or organism from organism.

"**Purchaser(s)**" means the organization purchasing and receiving ATCC Material pursuant to the ATCC Sales Order and this MTA.

"**Transferee**" means an employee or contractor that is a principal scientist or researcher at another organization or at Purchaser's organization. For the avoidance of doubt, Transferee specifically does NOT include any multi-user biological material core facilities or repositories to allow for the creation and provision of standardized reference materials derived from Biological Materials.

"**Trademarks**" means all trade name, trademark and service mark rights, whether registered or not, now owned or hereafter acquired, and the entire goodwill of the business of ATCC connected with and symbolized by such marks, including, without limitation, ATCC®, ATCC catalog marks, and ATCC Licensed Derivative®.

"**Unmodified Derivative(s)**" means substances created by Purchaser which constitute an unmodified functional subunit or product expressed by ATCC Material. By way of nonlimiting example, Unmodified Derivatives include: purified or fractionated subsets of the ATCC Material, proteins expressed by DNA/RNA supplied by ATCC or monoclonal antibodies secreted by a hybridoma cell line.

Scope of Use

ATCC Material and Progeny: ATCC Material and Progeny **may only be used by** Purchaser's Investigator for research purposes and only in Investigator's laboratory.

Modifications and Unmodified Derivatives: Purchaser's Investigator may make, use and transfer Modifications and Unmodified Derivatives only as follows:

- Purchaser's Investigator's Non-Commercial Use Research Project. Modifications and Unmodified Derivatives may only be made and used by Purchaser's Investigator for his/her research project(s) in Purchaser's Investigator's laboratory. Except as provided below Purchaser may only transfer such Modifications and Unmodified Derivatives, subject to any Contributor rights and CAR restrictions, to CROs and only for purposes related to Purchaser's Investigator's research project, and any such CRO must agree to be bound by the terms and conditions of this MTA as if a Purchaser hereunder and to not further transfer such materials. No subsequent transfer or Commercial Use of such materials is permitted without ATCC's written permission. Upon completion of any contracted research, Purchaser shall require CRO to either destroy materials or return them to Purchaser's Investigator.
- Non-Commercial Use Collaborative Research Project. Except as provided above Purchaser may only transfer Modifications and Unmodified Derivatives, subject to any Contributor rights and CAR restrictions, to Transferee(s) in Purchaser's Investigator's collaborative research project, so long as such Transferees agree to be bound by the terms and conditions of this MTA as if a Purchaser hereunder and to not further transfer such materials. For purposes of clarity, collaborative research project(s) shall not include any Commercial Use. No subsequent transfer of such materials is permitted without ATCC's written permission. Upon completion of any collaborative research project, the Purchaser shall require Transferee to either destroy such materials or return them to Purchaser's Investigator. Collaborative research projects include, but are not limited to Industry Sponsored Academic Research but permitted use hereunder extends only to basic and discovery research related to, directly under, or in direct collaboration with Purchaser's Investigator's research project. If parties working on a collaborative research project need to obtain Progeny, they should obtain ATCC Material directly, as ATCC Material and Progeny may not be transferred.

If such materials are transferred, Purchaser agrees to provide written notice to ATCC of any such transfer within a reasonable period after such transfer following the instructions available at: www.atcc.org/transfer so that ATCC may maintain a chain of custody of such material. Purchaser assumes all risk and responsibility in connection with the transfer of such materials.

Except as specifically provided in this section, Purchaser shall not distribute, sell, lend or otherwise make available or transfer to a person other than the Purchaser's Investigator or an entity not party to this MTA, the Biological Material, as defined above, for any reason, without ATCC's prior written agreement. Purchaser assumes all risk and responsibility in connection with the receipt, handling, storage, disposal, transfer, and Purchaser's and its Transferees' use of, the Biological Materials including without limitation taking all appropriate safety and handling precautions to minimize health or environmental risk.

Any Commercial Use of the Biological Material is strictly prohibited without ATCC's prior written consent. Purchaser acknowledges and agrees that Purchaser's use of certain Biological Material may require a license from a person or entity not party to this MTA, or be subject to restrictions that may be imposed by a person or entity not party to this MTA ("Third Party Terms"). To the extent of ATCC's knowledge of the existence of any such applicable rights or restrictions, ATCC will take reasonable steps to identify the same, either in ATCC's catalog of ATCC Material and/or through ATCC's customer service representatives, and to the extent they are in the possession of ATCC, ATCC shall make information regarding such Third Party Terms reasonably available for review by Purchaser upon request. Purchaser expressly acknowledges that if there is a conflict between this MTA and the Third Party Terms, the Third Party Terms shall govern. Use of the Biological Materials may be subject to the intellectual property rights of a person or entity not party to this MTA, the existence of which rights may or may not be identified in the ATCC catalog or website, and ATCC makes no representation or warranty regarding the existence or the validity of such rights. Purchaser shall have the sole responsibility for obtaining any intellectual property licenses necessitated by its possession and use of the Biological Materials.

The Biological Materials are not intended for use in humans. Purchaser agrees that Biological Materials designated as biosafety level 2 or 3 constitute known pathogens and that other Biological Materials not so designated may be pathogenic under certain conditions. Purchaser acknowledges that all purchases of ATCC Materials that are received under a CAR are governed by the terms and conditions of the CAR.

Identification of Modifications and Unmodified Derivatives in Transfers

Purchaser's Investigator shall assign and use its own naming scheme when transferring Modifications and Unmodified Derivatives to any Transferees and CROs. Purchaser's Investigator shall identify such Modifications and Unmodified Derivatives as having been tested or made using ATCC Material, and except as provided in the "Intellectual Property; Identification" section of this MTA, Purchaser's Investigator may not identify ATCC in any other context or otherwise use any ATCC Trademarks. By way of nonlimiting example, Purchaser may state that the Modification or Unmodified Derivative was tested or made using the ATCC Material identified with catalog number ATCC® (ATCC Catalog Number)™.

Warranty; Warranty Disclaimer

ATCC warrants that (a) cells and microorganisms included in the ATCC Material shall be viable upon initiation of culture for a period of thirty (30) days after shipment thereof from ATCC and (b) any ATCC Material other than cells and microorganisms shall meet the specifications on the applicable ATCC Material product information sheet, certificate of analysis, and/or catalog description until the

expiration date on the applicable ATCC Material's product label (such thirty (30) day period, or period until the expiration date, referred to herein as the "Warranty Period"). Purchaser's exclusive remedy, and ATCC's sole liability, for breach of the warranties set forth in this paragraph is for ATCC to, at ATCC's sole option, either (i) refund the fee paid to ATCC for such ATCC Material (exclusive of shipping and handling charges), or (ii) replace the ATCC Material. The warranties set forth in this paragraph apply only if Purchaser handles and stores the ATCC Material as described in the applicable ATCC Material product information sheet. To obtain the exclusive remedy, Purchaser must report the lack of viability or non-conformation to specifications to ATCC's Technical Service Department within the applicable Warranty Period. Any expiration date specified on the ATCC Material shipment documentation states the expected remaining useful life, but does not constitute a warranty or extend any applicable Warranty Period. **Except as expressly provided above, the ATCC Material and any technical information and assistance provided by ATCC are provided as-is, without warranties of any kind, express or implied, including but not limited to any implied warranties of merchantability, fitness for a particular purpose, typicality, safety, accuracy and/or non-infringement.**

Compliance with Laws

Purchaser is solely responsible for, and shall ensure, compliance with all foreign and domestic, federal, state and local statutes, ordinances and regulations applicable to use of the Biological Material by Purchaser or its Transferees. Purchaser is solely responsible for obtaining all permits, licenses or other approvals required by any governmental authority in connection with Purchaser's and its Transferees' receipt, handling, storage, disposal, transfer and use of the Biological Materials. Without limiting the generality of the foregoing, any shipment of Biological Materials to countries outside the United States must comply with all applicable foreign and U.S. laws, including the U.S. export control laws and related regulations. Distribution by ATCC of Budapest Treaty patent deposits are made pursuant to, and in compliance with, all applicable laws and regulations, including the Budapest Treaty and related 37 C.F.R. provisions. If there is any conflict between the terms of this MTA and any applicable law or regulation with respect to ATCC Material that is supplied hereunder by ATCC from the stock of a Budapest Treaty deposit, then the terms of the applicable law or regulation shall govern.

Indemnification

If Purchaser is a Federal or State non-profit organization or foreign organization that is prohibited by law from entering into the indemnification obligation set forth in the subsequent paragraph:
Purchaser assumes all liability for any and all third party claims, losses, expenses and damages, including reasonable attorneys' fees (collectively "Claims") arising out of or relating to Purchaser's and its Transferees' use, receipt, handling, storage, transfer, disposal and other activities relating to Biological Materials, provided that Purchaser's liability shall be limited to the extent that any such Claim arises out of ATCC's gross negligence or willful misconduct, and provided further that if the Purchaser is the U.S. federal government or a state institution or a foreign equivalent organization such Purchaser assumes such liability only to the extent provided under the Federal Tort Claims Act, 28 U.S.C. §§ 2671 et seq. or under equivalent applicable State or foreign law.

If Purchaser is a for-profit organization or a private non-profit organization:
Purchaser hereby agrees to indemnify, defend and hold harmless ATCC and its Contributors against all third party claims, losses, expenses and damages, including reasonable attorneys' fees (collectively "Claims") arising out of or relating to Purchaser's and its Transferees' use, receipt, handling, storage, transfer, disposal and other activities relating to Biological Materials, provided that Purchaser's liability shall be limited to the extent that any such Claim arises out of ATCC's gross negligence or willful misconduct. All non-monetary settlements of any such Claims are subject to ATCC's prior written consent, such consent not to be unreasonably withheld.

Limitation of Liability

To the extent permitted by law, in no event will ATCC or its Contributors be liable for any indirect, special, incidental or consequential damages of any kind in connection with or arising out of the MTA or Biological Materials (whether in contract, tort, negligence, strict liability, statute or otherwise) even if ATCC has been advised of the possibility of such damages. In no event shall ATCC's cumulative liability to the Purchaser exceed the fees paid by Purchaser under this MTA and the applicable ATCC Sales Order for the twelve (12) month period preceding the date of the event giving rise to the claim. Purchaser agrees that the limitations of liability set forth in this MTA shall apply even if a limited remedy provided hereunder fails of its essential purpose.

Intellectual Property; Identification

As between the parties, ATCC and/or its Contributors shall retain ownership of all right, title and interest in the Biological Materials. Purchaser retains ownership of: (a) Modifications (except that, as between the parties, ATCC retains ownership rights to ATCC Material incorporated therein) and (b) those substances created through the use of Biological Material, but which do not contain or constitute Biological Material. Notwithstanding the foregoing, Purchaser acknowledges and agrees that the Biological Material is subject to the restrictions noted in the "Scope of Use" section above. Purchaser agrees and shall ensure Transferee agrees to acknowledge ATCC and any Contributor indicated by ATCC as the source of the Biological Material in all research, academic or scholarly publications and in patent applications that reference the Biological Material. If required by the Contributor of the ATCC Material, ATCC may inform the Contributor of Purchaser's identity. Purchaser explicitly acknowledges that all Trademarks are the exclusive property of ATCC and Purchaser expressly agrees not to use the Trademarks without ATCC's prior written agreement.

Miscellaneous

Any disputes arising under this Agreement shall be tried exclusively in the United States District Court for the Eastern District of Virginia or if subject matter jurisdiction does not exist in that court, then in the state courts of Virginia for Prince William County, and Purchaser hereby expressly consents to, submits to and waives any objection to the jurisdiction of such courts. If Purchaser is a Federal or State non-profit organization or a foreign public organization then any disputes arising under this Agreement shall be tried exclusively in a court of competent jurisdiction.

Purchaser agrees that any breach of this Agreement, including but not limited to any breach of the scope of use provisions of this Agreement, will entitle ATCC to immediately cease without notice to Purchaser further shipments of ATCC Material and may create such irreparable injury as to entitle ATCC to seek temporary restraining orders and other preliminary or permanent injunctive relief in addition to all other equitable and legal remedies that may be afforded under US or foreign laws.

Purchaser may not assign or otherwise transfer this MTA or any rights or obligations under this MTA, whether by operation of law or otherwise. Any such attempted assignment or transfer will be void and of no force or effect. This MTA, including all documents incorporated herein by reference, constitutes the entire agreement between ATCC and Purchaser with respect to the Biological Material and supersedes all previous agreements or representations (whether written or oral) between ATCC and Purchaser relating to the same subject matter. This MTA may not be modified, waived or terminated except in writing and signed by the parties hereto. No term or provision contained herein shall be deemed waived and no breach excused unless such waiver or consent shall be in writing and signed by the parties. If any provision of this MTA is for any reason found to be unenforceable, the remainder of this Agreement will continue in full force and effect. None of the provisions of this MTA are intended to create, nor shall be deemed or construed to create, any relationship between ATCC or Purchaser other than that of independent entities contracting with each other hereunder solely for the purpose of effecting the provisions of this MTA.

By signing below, the duly authorized representative of each party acknowledge that they have read and understood and agree to the terms and conditions set forth in this Material Transfer Agreement, as evidenced by their signatures below.

For ATCC

By: [signature]

Name: Stewart N. Davis

Title: Director, Intellectual Property and Licensing

For PURCHASER

By: Cinzia Marchese

Name: Cinzia Marchese

Title: Full Professor

Date: 05/24/2017

Any correspondence concerning the ATCC Material Transfer Agreement should be addressed to ATCC, Attention: Director, IP & Licensing, Corporate Development, P.O. Box 1549, Manassas, VA 20108, Phone: (703) 365-2700 or contact us at licensing@atcc.org
